# Identification and prediction of developmental enhancers in sea urchin embryos

**DOI:** 10.1186/s12864-021-07936-0

**Published:** 2021-10-19

**Authors:** César Arenas-Mena, Sofija Miljovska, Edward J. Rice, Justin Gurges, Tanvi Shashikant, Zihe Wang, Sevinç Ercan, Charles G. Danko

**Affiliations:** 1 College of Staten Island, The City University of New York (CUNY), Staten Island, NY 10314 USA; 2 Programs in Biology and Biochemistry, The Graduate Center, CUNY, New York, NY 10016 USA; 3 Department of Biology, New York University, New York, NY 10003 USA; 4 Baker Institute for Animal Health, College of Veterinary Medicine, Cornell University, Ithaca, NY 14853 USA; 5 Department of Biological Sciences, Carnegie Mellon University, Pittsburgh, PA USA; 6 Center for Genomics and Systems Biology, New York University, New York, NY 10003 USA; 7 Department of Biomedical Sciences, College of Veterinary Medicine, Cornell University, Ithaca, NY 14853 USA

**Keywords:** Cis-regulatory element, Enhancer prediction, Developmental gene regulation

## Abstract

**Background:**

The transcription of developmental regulatory genes is often controlled by multiple cis-regulatory elements. The identification and functional characterization of distal regulatory elements remains challenging, even in tractable model organisms like sea urchins.

**Results:**

We evaluate the use of chromatin accessibility, transcription and RNA Polymerase II for their ability to predict enhancer activity of genomic regions in sea urchin embryos. ATAC-seq, PRO-seq, and Pol II ChIP-seq from early and late blastula embryos are manually contrasted with experimental *cis-*regulatory analyses available in sea urchin embryos, with particular attention to common developmental regulatory elements known to have enhancer and silencer functions differentially deployed among embryonic territories. Using the three functional genomic data types, machine learning models are trained and tested to classify and quantitatively predict the enhancer activity of several hundred genomic regions previously validated with reporter constructs in vivo.

**Conclusions:**

Overall, chromatin accessibility and transcription have substantial power for predicting enhancer activity. For promoter-overlapping cis-regulatory elements in particular, the distribution of Pol II is the best predictor of enhancer activity in blastula embryos. Furthermore, ATAC- and PRO-seq predictive value is stage dependent for the promoter-overlapping subset. This suggests that the sequence of regulatory mechanisms leading to transcriptional activation have distinct relevance at different levels of the developmental gene regulatory hierarchy deployed during embryogenesis.

**Supplementary Information:**

The online version contains supplementary material available at 10.1186/s12864-021-07936-0.

## Background

Transcriptional regulatory elements (TREs) [1] are the primary drivers of differential gene expression during metazoan development [2–4]. Whereas promoters are TREs easily found by association with the transcription start sites (TSSs) of genes, the identification and functional characterization of TREs distal to TSSs (enhancers and silencers) remains challenging. Traditionally, enhancers have been considered the modulators of distal transcription at core promoters (promoters thereafter), which integrate inputs from enhancers and ‘proximal promoters’ to initiate local transcription [5]. However, this exclusive functional distinction has been blurred by recent evidence that reveals local transcription initiation at enhancers and promoters that modulate the transcription of some other promoters [1, 2, 5]. Nevertheless, distinct sequences and chromatin features associate with the prevalence of enhancer and promoter activities among TREs [6]. The expression of inducible genes in unicellular organisms is oftentimes driven by regulatory sequences proximal to the core promoter [2, 5]. In contrast, the oftentimes complex expression of developmental regulatory genes (that is, transcription and signaling factors) is primarily controlled by distal regulatory elements [2, 7]. Despite their relevance, only a few *cis-*regulatory modules (CRMs) that constitute the essential transcriptional nodes of developmental gene regulatory networks are functionally understood [8]. Various high-throughput reporter assay with particular advantages and limitations [9] allow genome-wide testing of enhancer activity [10], and, despite recent progress, most TREs remain largely uncharted [6, 11]. Histone marks such as H3K27ac [12, 13], chromatin accessibility [14] or transcription initiation [15] facilitate the identification of active enhancers. However, the systematic evaluation of the predictive power and redundancy of these genomic marks remains limited [13, 16]. Enhancer transcription may facilitate enhancer activity prediction because it represents the end product, possibly subsequent in most cases to chromatin accessibility set in part by H3K27 acetylation, and because it correlates between enhancers and their target promoters [17–19]. In particular, we are interested in the predictive value of enhancer transcription estimated by the analysis of the transcription run-on assay PRO-seq [20], which detects the differential location of paused and elongating RNA Pol II associated with distinct transcriptional regulatory states [17].

Several experimental advantages have facilitated the exhaustive reconstruction of developmental gene regulatory networks (GRNs) in sea urchin embryos [21–23]. The analysis of topological GRN models reveals an uneven distribution of regulatory sub-circuit motifs along the GRN hierarchy sequentially deployed during sea urchin embryogenesis [4]. Accordingly, the structure and Boolean logic of the TREs serving the nodes of these sub-circuits changes during development too [22]. In sea urchins, enhancer and silencer activities of TREs can be tested by lack of function in bacterial artificial chromosome (BAC) reporter constructs microinjected into zygotes [24, 25], or by gain of function in much smaller plasmid reporters, which oftentimes use heterologous promoters [26]. These exogenous reporters replicate along with the genome [27, 28], with the much larger than plasmids BACs providing a closer approximation to the natural genomic context and more faithfully reproducing the endogenous expression. In addition, BACs maintain endogenous promoters in the context of gene-reporter translational fusions. Despite these advantages, the identification and testing of developmental TREs remains challenging due to the low throughput of existing experimental approaches.

**Fig. 1:**
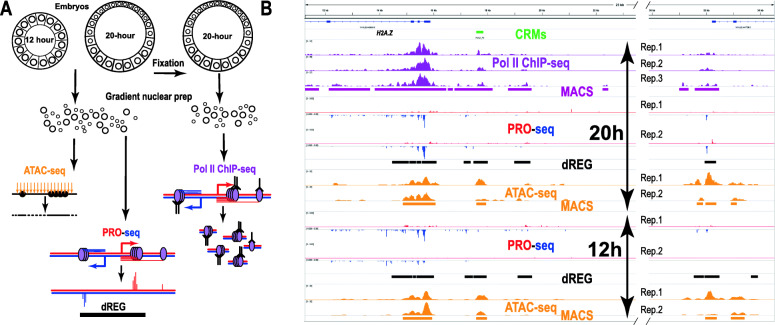
ATAC-seq, PRO-seq and Pol II ChIP-seq are used for the identification of TREs. **A** Experimental outlines of the 3 genomic profiles used. **B** IGV browser snapshot of replicate genomic profiles at the *H2A.Z* locus, a highly expressed gene [36], left, which also includes a gene expressed at lower levels, right side. Number of 3′ end reads per million of PRO-seq run-on transcripts are shown for the plus and minus strands. PRO-seq peaks mark transcriptional pause sites. MACS peak and dREG TRE predictions for the combined data sets are shown underscoring each genomic profile. The CRM panel underscores a genomic region with enhancer activity tested by deletion in large reporter constructs [25]. PRO-seq and ATAC-seq profiles are set to the same scale between 12 and 20 h stages, with the range indicated between brackets at the beginning of each track

Evolutionary sequence conservation has been routinely used for the identification of potential TREs in sea urchins [4], although conservation is not informative about the stage of regulatory activity, and the obscure cause of diverse evolutionary rates for regulatory sequences [29, 30] raises great uncertainty regarding false positive and negative rates. As in other model systems, chromatin accessibility also facilitates the identification of candidate enhancers in sea urchin embryos [25, 31]. In addition, a parallel reporter method enabled the enhancer activity test of several hundred CRMs associated with 37 developmental regulatory genes during sea urchin development [32, 33]. We used this functional quantification to train and test machine learning model predictors of developmental enhancer activity from various genomic profiles: chromatin accessibility estimated by ATAC-seq [34], RNA Polymerase II (Pol II) distribution, which has been also associated with active enhancers [35], was detected by ChIP-seq, and transcription initiation by PRO-seq (Fig. 1A). Our analysis reveals that chromatin accessibility and transcription both enable enhancer activity prediction, and that the predictive power of these genomic profiles declines during development for the subset of promoter proximal TREs, suggesting a sequence of regulatory shifts at different levels of the gene regulatory hierarchy that is deployed during development.

## Results

### Genomic distribution of chromatin accessibility, pol II and transcription

Chromatin accessibility, transcription and the distribution of RNA Pol II using ChIP-seq were used for the identification of candidate developmental enhancers in 20 h sea urchin embryos (Fig. 1A), and the best performing predictors, ATAC- and PRO-seq, see later, were also characterized in 12 h embryos. The genomic profile of 3′ end transcripts identified by PRO-seq was analyzed with dREG (Fig. 1A), a support vector regression tool trained to identify TREs associated with active chromatin marks using the shape of transcription [15]. In 12 and 20 h embryos, dREG identified 43,912 and 56,753 TRE predictions or “peaks”, respectively, while a total of 238,838 and 258,515 ATAC-seq peaks, respectively, were called by MACS (QC reports in supplementary information). In 20 h embryos, 554,846 Pol II ChIP-seq peaks were called.

**Fig. 2:**
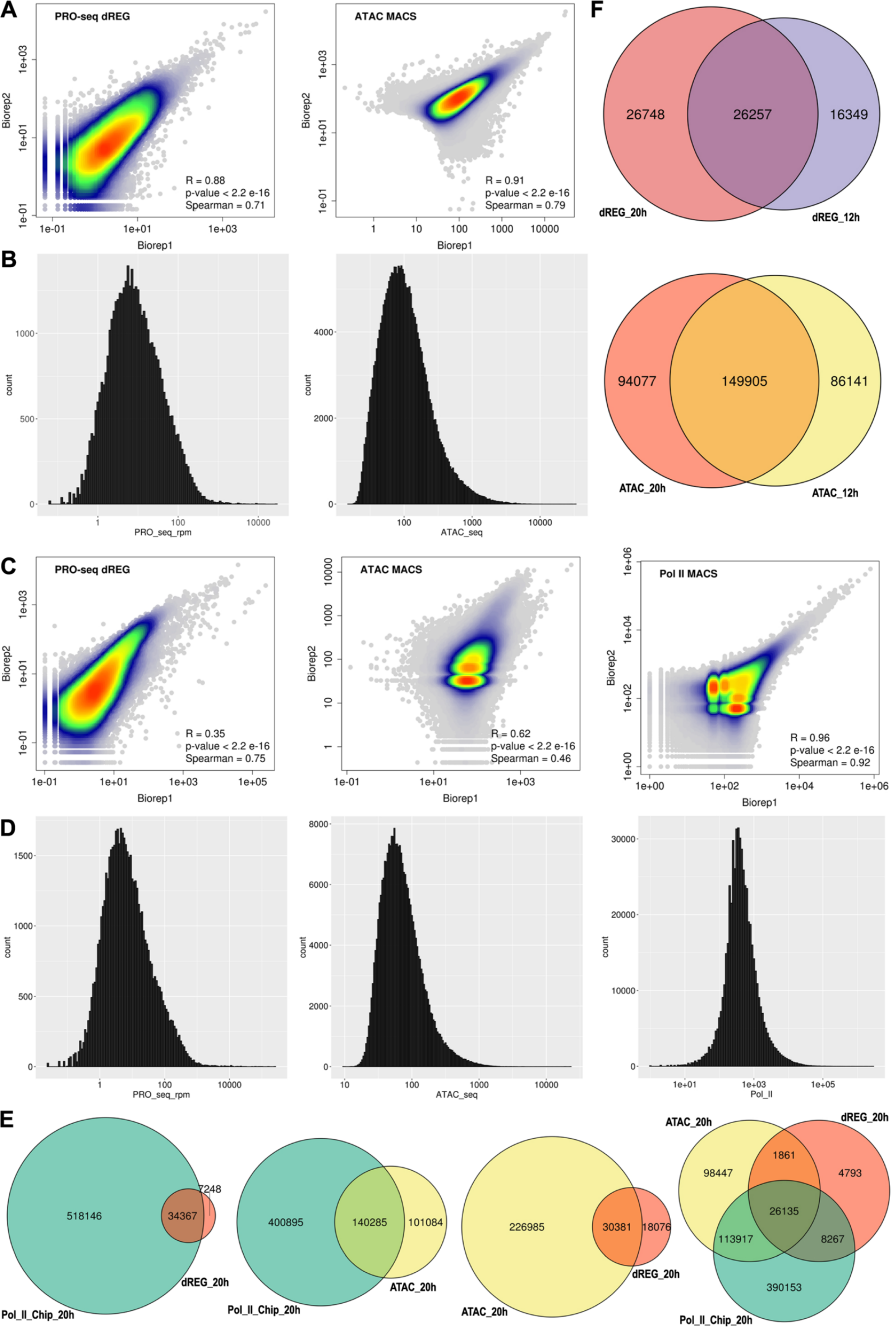
Genome-wide PRO-, ATAC- and ChIP-seq analysis. **A** Distribution of signal intensity and reproducibility estimation between distinct biological replicates for the different data sets in 12 h embryos. Overlap of points indicated by the color gradient. **B** Histograms of the number of reads per peak call for the different data sets in **A**. **C** Distribution of signal and reproducibility in 20 h embryos. **D** Histograms of the number of reads per peak call for the different data sets in **C**. **E** Venn diagrams of the overlap between ATAC and Pol II ChIP peak calls, and dREG predicted TREs in 20 h embryos. **F** Venn diagrams of the overlap of ATAC and dREG peak calls between stages

The dynamic range of the read distribution at peak calls expands several orders of magnitude for the three functional genomic data types (Fig. 2B and D). The Pearson correlation of total PRO-seq reads at dREG peak calls of biological replicates is higher for 12 h embryos (R = 0.88, *p*-value < 2.2 × 10^− 16^, Spearman = 0.71, Fig. 2A) than for 20 h embryos (R = 0.35, p-value < 2.2 × 10^− 16^, Spearman = 0.75, Fig. 2C). Similarly, higher correlation for ATAC-seq profiles at MACS peak calls is found for 12 h embryos (R = 0.91, p-value < 2.2 × 10^− 16^, Spearman = 0.79, Fig. 2A) relative to 20 h embryos (R = 0.62, p-value < 2.2 × 10^− 16^, Spearman = 0.46, Fig. 2C). The low correlation of PRO-seq biological replicates may be due in part to inherent batch heterogeneity associated with seasonal and genetic variability in the wild populations from which the embryos where obtained. This natural variation may shift the relative timing of major transcriptional regulatory changes during and prior to the 20 h embryo stage, as previously reported [36, 37], along with the associated histone modification and chromatin accessibility signals (Fig. 2).

Interestingly, there is less variability among Pol II ChIP-seq biological replicates (R = 0.96 to 0.94, with *p*-values < 2.2 × 10^− 16^, Fig. 2C). Nevertheless, the biological or technical source of the variation among the different marks could not be resolved in this study, because different embryo batches, sometimes from different seasons, were used. In addition, for the 20 h ATAC-seq data, a distinct nuclear extraction protocol for one of the replicates [25] may have contributed to some technical variability. Given these caveats no quantitative genome-wide comparison of the different genomic profiles between stages is performed, but only primarily qualitative comparisons among stages based in peak calls (Fig. 2F and Fig. S1 F). However, despite the higher variability of the PRO-seq and ATAC-seq 20 h data sets, generally similar signal profiles are seen among biological replicates in both developmental stages, as illustrated at the *H2A.Z* locus [25] (Fig. 1B). Similar reproducibility trends are observed at promoters and CRMs (Fig. S1 A-D), with much higher replicate correlations for the subset of CRMs that are the primary target of this study (CRMs hereafter) (Fig. S1 B and D).

Genome-wide, most dREG peaks overlap Pol II peaks (Fig. 2E), as expected. However, because much of Pol II is found in the body of transcribed genes, the majority of pol II peak calls did not overlap with dREG predictions. About 40% of ATAC-seq peaks do not overlap Pol II peaks, and about 90% of ATAC-seq peaks do not overlap dREG predictions, revealing that a substantial fraction of chromatin-accessible regions do not associate with RNA Pol II or transcription initiation detected using dREG, which depends on local transcription initiation profiles. Similar overlapping trends are observed in the CRMs target of this study, with a much larger fraction of ATAC peaks overlapping Pol II peaks, dREG predictions and both (Fig. S1 E), possibly in association with a transcriptional regulatory enrichment in the CRM data set, which is strongly biased for evolutionary sequence conservation [33].

The distinct peak numbers and particular overlaps among the three genomic assays anticipate distinct contributions and/or the requirement of combinatorial analysis for the prediction of distal TREs. Globally, about 42 and 50% of the 12 and 20 h dREG peaks are stage specific, respectively (Fig. 2F), while 36 and 38% of the 12 and 20 h ATAC peaks are stage specific, respectively (Fig. 2F) . However, for peaks overlapping CRMs (Fig. S1 F), the majority of ATAC and dREG peaks present in the 12 h stage remain in the 20 h stage, but a much larger proportion of dREG peaks than ATAC peaks are 20 h specific, 60% versus 30%. This reveals that during the 12 to 20 h transition there is a general increase of transcription initiation and pause at developmental TREs while accessibility, estimated by peak calls, is more constant. This suggests that increased accessibility of developmental enhancers generally precedes transcriptional output of developmental TREs.

### Validation and evaluation of functional genomic marks for the identification of developmental TREs

**Fig. 3:**
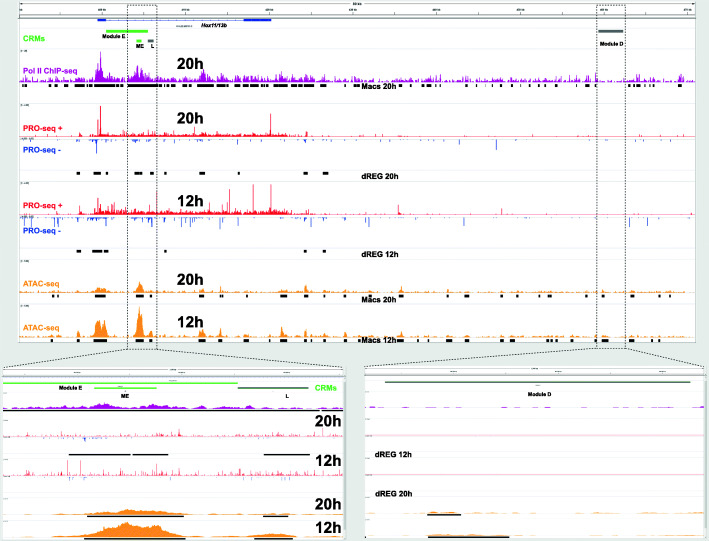
ATAC-, Pol II ChIP- and PRO-seq sea urchin embryos at the *SpHox11/13b* locus. For ATAC- and PRO-seq, the scale in reads per million at the start of each track is maintained at the same range between states and equal between plus and minus strands. The whole region was scanned for enhancer activity by overlapping 3–5 Kb reporter constructs [24], only active CRMs are indicated, in green those active in both stages, and in gray those inactive or with unknown activity in these stages as indicated in the text

We manually contrasted our functional genomic data sets with previous experimental *cis*-regulatory analyses in order to explore how they could facilitate the identification of active developmental TREs. TRE necessity for the control of endomesoderm transcription factor *SpHox11/13b* developmental expression has been characterized by deletion from BAC reporters, and TRE sufficiency by plasmid reporter constructs testing an overlapping array of genomic regions that scan the entire locus [24]. ATAC-seq peaks underscore regulatory element ME in 12 and 20 h embryos but only dREG highlights ME in 20 h embryos (Fig. 3), which corresponds to the stage of higher ME reporter activity [24].

This pattern of 20 h specific dREG activity follows the general ATAC and dREG stage prevalence trend in the proximity of regulatory elements, a generally constant number of ATAC peaks and increased number of dREG peaks during the later blastula stage (Fig. S1 F). Module ME has been demonstrated to be both necessary by deletion in BAC reporters and sufficient in plasmid reporters [24] to drive the embryonic *SpHox11/13b* expression profile, which is spatially dynamic and increases during the 12 to 20 h transition [38]. Like many other early embryo TREs, ME has distinct enhancer and silencer functions in different embryonic territories. In 12 and 20 h embryos, module ME responds to spatially restricted vegetal *wnt* signaling by enhancing transcription in the endomesoderm and endoderm territories, and by silencing transcription in the ectoderm and the mesoderm, where the *wnt* pathway remains and becomes inactive, respectively [24]. Therefore, the whole embryo genomic profiles derive from both enhancer and silencer activities from different territories.

DNA binding sites for transcription factor TCF are required for the *wnt* signal dependent enhancer and silencer functions of element ME. There is an increase in the transcriptional pause at the 20 h embryo *SpHox11/13b* promoter relative to the 12 h stage (Fig. 3), which could correspond with its ME transcriptional silencing in the ectoderm and mesoderm [24]. Interestingly, the cofactor of TCF, groucho, implements silencing by pause in Drosophila embryos [39], which may represent an evolutionarily conserved function in sea urchins. Module D, in isolation, drives unrestricted reporter expression in 15 and 18 h embryos that can be dominantly silenced by module ME when placed in the same reporter construct [24]. Module D is inactive in 6 and 21 h embryos, leaving uncertain its activity in 12 h embryos. There are ATAC-seq peak calls with relatively low signal within Module D in both stages, but no dREG peaks (Fig. 3). Thus, module D lacks silencing functions and dREG peak calls.

Module D was not deleted in BACs and therefore its endogenous function remains uncertain [24]. Finally, element L, which drives reporter activity at later stages, was undetectable with reporter constructs in 15 and 24 h embryos [24], dREG marks regulatory element L in 20 h embryos but not in 12 h embryos, while ATAC detects this regulatory element in both stages. In this case, the dREG peak calls and associated pausing in module L of 20 h embryo may correspond with its priming for subsequent activation during later embryonic and larval stages.

Similar unbiased tiling array reporter scan was performed for the regulatory elements controlling the expression of transcription factor *onecut* [40]. During pregastrular stages, *onecut* zygotic transcript levels reach a minimum soon after the 12 h stage and peak at 20 h, around the time when its restricted oral-aboral boundary expression is stablished [40]. Analogous to *SpHox11/13b* module ME, distal regulatory module Intron-D of *onecut* integrates enhancer and silencer functions that are necessary and sufficient to recapitulate the expression of this transcription factor [40]. Likewise, ATAC-seq peak calls underscore Intron-D in both stages, but only 20 h dREG peak calls highlight Intron-D, coincident with an augment of pause at the *onecut* promoter (Fig. S2). Thus, both in *SpHox11/13b* ME and *onecut* Intron-D PRO-seq signals may correspond to a blend of transcriptional activation and silencing functions in different embryo regions. Only ATAC-seq peaks intersect *onecut* Intron-C module, which is inactive in 20 h embryos (Fig. S2). Thus, similar to module L of *SpHox11/13b,* the ATAC-seq peak call does not correspond with enhancer activity.

There are far more ATAC-seq peak calls than dREG predictions at both loci (Fig. 3 and Fig. S2), along with the general genomic trend (Fig. 2E). Both loci were scanned by a comprehensive reporter tiling scheme to test the entire regions for enhancer activity in an unbiased manner [24, 40]. Remarkably, dREG TRE predictions correspond closely with regulatory elements experimentally mapped to their minimum range (Fig. 3 and Fig. S2). However, most dREG predictions do not match CRM enhancer reporter activity (Fig. 3 and Fig. S2), and even a higher proportion of ATAC peaks do not correspond with enhancer-active CRMs. The distribution of Pol II ChIP peaks is even broader, particularly at introns, which are prone to contain TREs (Fig. 3 and Fig. S2), and, therefore, Pol II ChIP signal seems poorly suited for TRE predictions on its own. Manual analysis of other experimentally characterized transcriptional regulatory elements [41–43] generally confirms the trends outlined above (Fig. S2). Additional regions of the genome can be analyzed with the data sets deposited at the NCBI Gene Expression Omnibus [44], which include ATAC-, Pol II ChIP- and PRO-seq genomic profiles along with their associated peak calls. In summary, our manual analysis suggest that accessibility may have a looser correspondence with enhancer activity. In addition, although increased pause does not necessarily correspond with silencing, as it may be associated with increased release and elongation, the dual report of transcriptional elongation and pause by PRO-seq may correspond to enhancer and silencer activities differentially deployed in space, which is very common among early embryo regional specification TREs [4].

### Prediction of enhancer activity from chromatin accessibility and pol II

**Fig. 4:**
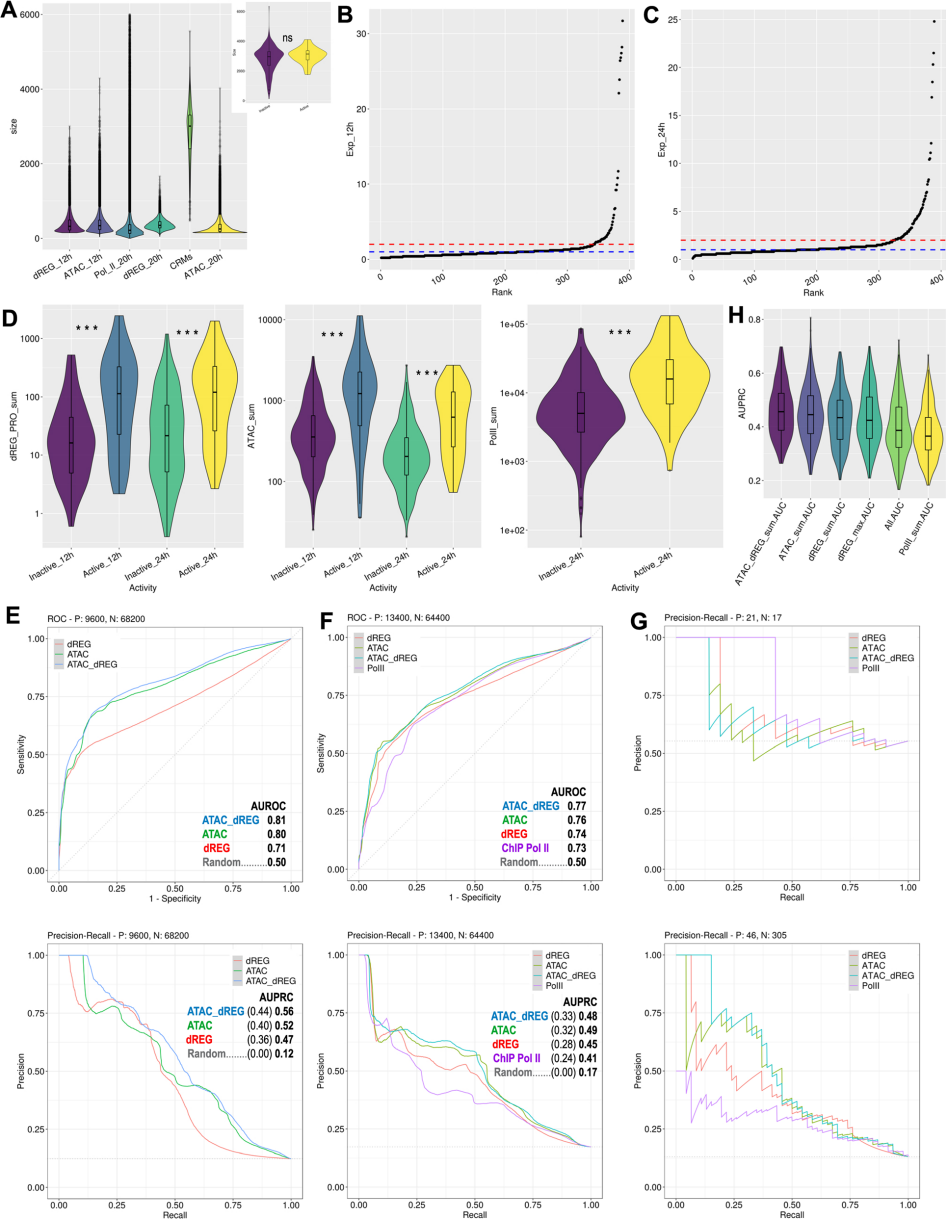
Modeling of CRM reporter activity from of ATAC-, Pol II ChIP- and PRO-seq. **A** Violin/box-plot of the ATAC, Pol II ChIP peak call and dREG TRE prediction sizes, and the 389 CRMs. The inset plots the size distributions of active and inactive CRMs, which is not significatively different. **B** and **C**, ranked CRM expression plot in 12 and 24 h embryos, respectively. The blue line at 1 marks the CRM expression level when it equals that of the basal-promoter reporter. The red line by the curve “elbow” marks the 2 fold above control chosen as the expression threshold. **D** Violin/box-plots of PRO-, ATAC-, and Pol II ChIP-seq significatively different signals between active and inactive CRMs in 12 and 20 h embryos. **E**, top, 12 h embryo Receiver Operating Characteristics (ROC) and, bottom, Precision-Recall Curves (PRC) of the logistic regression models trained and tested by 5 fold cross-validation repeated 200 times. Area Under the ROC (AUROC) and AUPRC as indicated for each model. Dotted lines mark random guess prediction performance, a mid-diagonal for ROC and a horizontal line at the fraction of active CRMs for PRC. The absolute AUPRC indicated in bold and the difference with random guess in parenthesis. **F** ROCs and PRCs in 20 h embryos. **G**, top, PRCs evaluating the enhancer activity predictions for the CRM promoter-overlapping data set of models trained with the entire 20 h CRM data set. Bottom, model predictions for the complementary, non-promoter overlapping data set. **H** Violin/box-plot of the AUPRC after cross-validation with different predictors, as indicated; All, includes the sum and max of the 3 genomic profiles allowing second order interactions among predictors; dREG-max, signifies the sum of the maximum values at dREG peaks

We decided to systematically test if machine learning models using chromatin accessibility, Pol II distribution and transcription initiation could predict the previously quantified enhancer activity of 389 CRMs (Supplementary Table 1) primarily selected for their evolutionarily sequence conservation [33]. We first tested a subset of reporters quantified at high temporal resolution using nanoString technology [32], but the very small number of inactive reporters was unsuitable for model training (results not shown). We therefore settled for a previous data set that measured reporter enhancer activity by qPCR and included 12 and 24 h time points [33]. Although this generated a mismatch with the 20 h stage examined in our genomic data, no major regulatory transitions have been identified for the majority of the genes involved during this 4 h period [45]. The average size of the 389 CRMs (2839 bp) is about one order of magnitude larger than the average of ATAC peaks (316 bp) Pol II peaks (338 bp), and dREG TRE predictions (373 bp) (Fig. 4A). Thus, in order to reduce confounding inputs to the CRMS that may not relate to TRE function, such as background transcription at introns, or background accessibility along the CRM span, the ATAC-, Pol II ChIP- and PRO-seq signals were computed at CRM regions overlapping peak calls and dREG predictions. CRMs were defined as active if they drove reporter expression twice above the basal promoter (Fig. 4B and C). CRM activity cannot be explained by CRM size because there is no significant size difference between active and inactive enhancers (Fig. 4A, inset, Wilconox *p*-value = 0.11). However, active CRMs in 12 and 24 h embryos have significatively higher PRO-, ATAC-, and Pol II ChIP-seq signals (Fig. 4D, *p*-values < 1.8 e-06).

Logistic regression classifiers trained and tested by 5 fold cross-validation repeated 200 times resulted in predictions significatively above random guess in both embryo stages (Fig. 4E and F). The performance of models using ATAC, dREG (PRO-seq reads at TRE dREG predictions), their combination (ATAC + dREG), and Pol II ChIP was slightly higher for ATAC + dREG models when evaluated by the Area Under the Receiver Operating Characteristic (AUROC) plot (Fig. 4E and F), which graphs the relation between true and false positive rates at different model prediction thresholds. However, the CRM expression data set is highly unbalanced, with about 10 times more CRMs reporting inactive than active enhancer activity (Fig. 4B and C), and, in these cases, Precision-Recall Curves (PRCs), which plot precision values along the range of true positive rates, provide a better discrimination metric for classifier evaluation [46].

When AUPRCs are used for model evaluation, more distinct model performances are obtained, particularly for the 20 h data sets (Fig. 4F, bottom). Individually, all assays perform much better than chance in both stages (Fig. 4E and F). The combination of ATAC and dREG predictors barely improves performance at some recall values (Fig. 4E and F, bottom), and similarly, Pol II ChIP signal does not facilitate better enhancer activity predictions alone (Fig. 4F) or in combination with other data sets (results not shown). The limited model improvement with combined data sets is perhaps expected given their substantial correlation, with Pearson’s correlation coefficients ranging from 0.6 to 0.8 between predictors of the same stage. Incorporation of other parameters such as peak summit value, did not improve any predictive models, as illustrated for dREG (Fig. 4H). As expected, lower model performance was also obtained when the functional genomic data were computed along the entire CRM instead of restricting the signal input to peak call windows (not shown). Optimization of other machine learning methods, such as random forest and support vector machine, did not improve classifier performance over logistic regression (not shown), likely reflecting the small size of the available data. In short, total ATAC-seq and PRO-seq signals at dREG peaks are the best predictors of active enhancer activity among the profiles tested in this study.

About half of the CRMs that overlap promoters are active in the reporter assays, indicating a high degree of enhancer activity from promoter-adjacent DNA in sea urchin embryos [33]. Nearly all these promoter-overlapping CRMs were previously shown to be active in both orientations [33], demonstrating bona fide enhancer activity. We further confirmed that the concurrent and divergent orientation of promoter-overlapping CRMs in reporter constructs were evenly represented in our data set and did not correspond with significant enhancer activity differences (Fig. S3), excluding the relevance of confounding effects due to transcription initiation at the CRMs followed by elongation into the reporter. The sizes of promoter-overlapping CRMs (Fig. 4A) suffice to include both distal and proximal TREs, including promoters. Interestingly, ATAC and dREG models trained with the entire data set (Fig. 4F) underperformed relative to Pol II ChIP based models in the prediction of enhancer activity of the promoter-overlapping CRM subset (Fig. 4G, top). In the complementary analysis, the Pol II ChIP model trained with the entire set further underperformed relative to ATAC and dREG models in the prediction of CRMs not overlapping promoters, while ATAC and dREG maintained performance similar to predictions with the entire set (Fig. 4G, bottom). The exclusion of the 41 promoter-overlapping CRMs from the training and testing data set decreased the prediction performance of all models in both stages (Fig. S4 A and B). Overall performance was broadly similar between ATAC and dREG models trained and tested with the promoter overlapping or non-overlapping in 12 h embryos (Fig. S4 A and C). In contrast, ATAC and dREG models trained with CRM-overlapping promoters failed to predict the activity of their hold out set and were outperformed by Pol II ChIP models in the 20 h data set (Fig. S4 D). All of the above, reveals that the enhancer activity predictive power of ATAC-seq and PRO-seq for promoter-proximal CRMs dramatically devalues during the 12 to 20 h transition.

The larger proportion of positive enhancers among CRMs that overlap promoters relative to CRMs not overlapping promoters, ~ 50% vs. ~ 13% in 20 h embryos, is not surprising given the bias for regulatory genes active during development of this data set [33] combined with the general trend of enhancers to be near their promoter targets [47]. The enhancer activity of promoters has precedents [48, 49] and it is perhaps not surprising for evolutionary reasons [2].

**Fig. 5:**
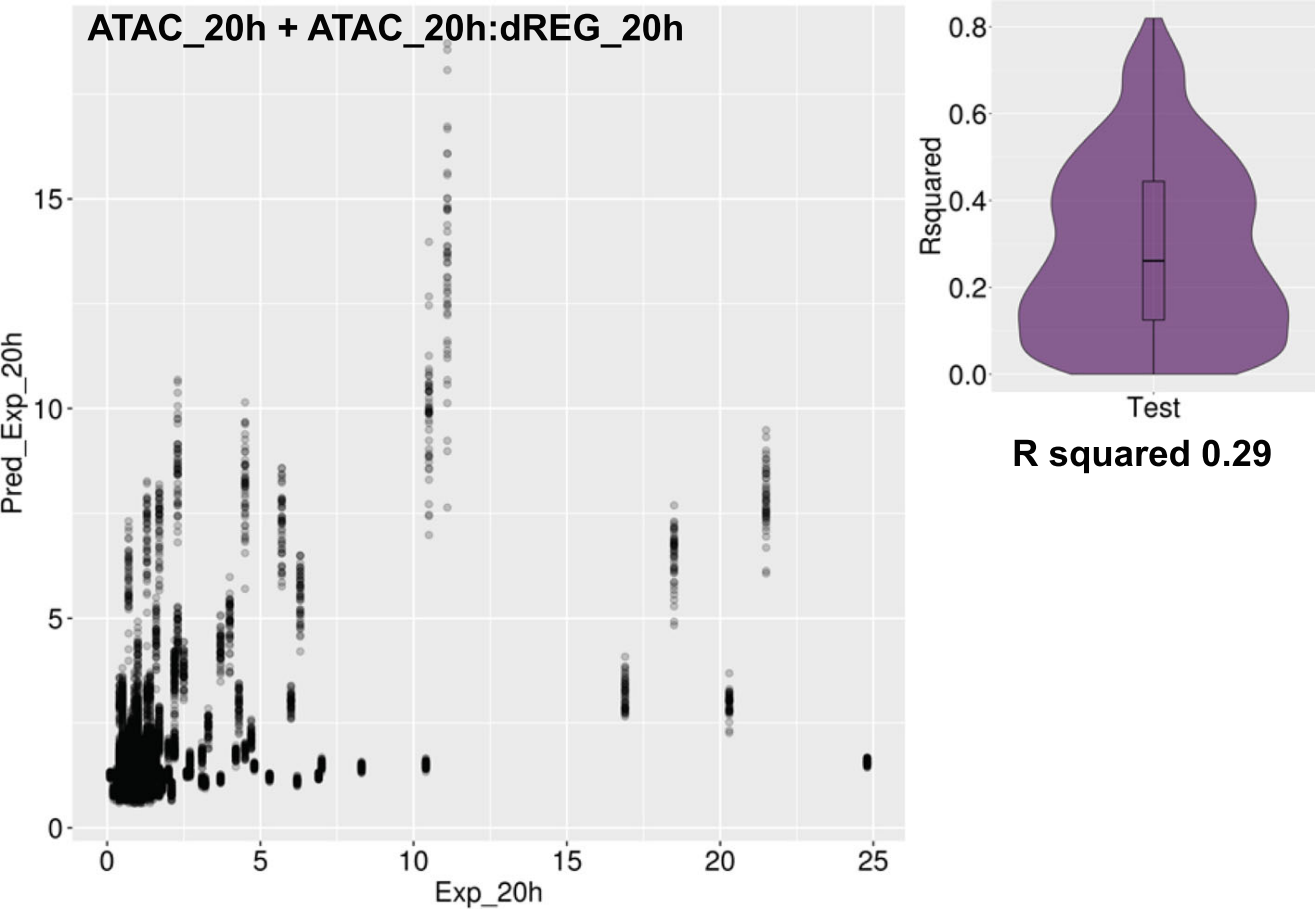
Quantitative prediction of enhancer activity from PRO-seq data. Plot of the hold-out predicted against the actual reporter expression of linear regression models using ATAC and PRO-seq signal at dREG predictions tested by five-fold cross-validation. Violin/Box-plot of R^2^ values, with the average indicated underneath

We tested if the functional genomic datasets could predict the levels of reporter enhancer activity of CRMs. In all cases, better linear regression model prediction was obtained with non-promoter overlapping CRM sets. The best performing model included the ATAC-seq plus the ATAC:dREG interaction, which explained about one third of the expression variation (average *R*^*2*^ = 0.29) in 20 h embryos (Fig. 5). ATAC-seq was a better predictor of enhancer activity in 20 h embryos relative to 12 h embryos (*R*^*2*^ = 0.26 versus *R*^*2*^ = 0.17, *p*-value < 2.2 e-16), and outperformed dREG in 20 h embryos (*R*^*2*^ = 0.17, p-value = 7.4 e-16) (Fig. S5). The difference in dREG model performances between stages or with ATAC-seq models in 12 h embryos was not significant due to the small size of the dataset. Nevertheless, the relative enhancer predictive power of ATAC-seq and PRO-seq is stage-dependent. The predicted value of most CRMs generally varies with the training set, as expected. However, there is a group of CRMs that are consistently and erroneously predicted as barely active (Fig. S5) due to their low signals in all assays (not shown). Highly active reporter constructs with low predictor signals may result from not uncommon miss-regulation outside the endogenous genomic context [4], which may cause ectopic expression, such as the one observed for *SpHox11/13b* module D [24], or it may reflect mismatches between the time points, especially at the 20 h stage.

## Discussion

Machine learning models identify ATAC- and PRO-seq as efficient predictors of the developmental enhancer activity of genomic regions previously validated by their reporter driven expression in sea urchin embryos (Fig. 4E and F). Further prediction improvements are expected after addressing some limitations and biases in of our experimental setup. First, the bulk functional genomic profiles of whole embryos represent a blend of several transcriptional states present in different territories. The unavoidable bias against enhancers only active in a few cells should nevertheless correspond with similarly biased CRM reporter expression levels. More problematic could be the confounding enhancer and silencer activities in different territories for the same element, which are common in developmental gene regulation [4, 50], as previously discussed in the context of *SpHox11/13b* and *onecut* (Fig. 3 and Fig. S2). Future single-cell ATAC-seq studies will increase the spatial resolution over whole embryo characterizations, such as the one presented here, which are nevertheless required for validation. To our knowledge, there is no single-cell approach for PRO-seq. However, single-cell resolution should be also developed for the CRM functional assays in order to fully overcome this limitation. Second, the mismatch between the size of TRE peak calls and CRMs tested is less than ideal (Fig. 4A). In most high-throughput reporter assays [10], the regulatory regions tested are usually smaller than the few hundred base pairs of common TREs, which represents one of the several limitations of enhancer activity evaluation by reporter constructs [10]. In contrast, the genomic regions tested in our data set are large (Fig. 4A) and often contain several ATAC and dREG peak calls, whose signals would possibly better match enhancer activity if individually tested. Third, the CRMs functionally tested are biased for evolutionary sequence conservation, which may exclude functional but fast evolving CRMs. Thus targeting the functional analysis to ATAC-seq and dREG peak call regions would be more suitable to analyze the predictive value of these genomic profiles. Fourth, inherent to most high throughput reporter assays, enhancer activity is tested with a heterologous promoter, allowing for the mismatch between functional genomic assays and reporter activity due to enhancer-promoter specificity [51].

Despite the tight match between dREG TRE predictions and CRMs experimentally narrowed down to the smallest functional regulatory elements in a generally unbiased manner (Fig. 3), our results reveal that PRO-seq has similar predictive power as ATAC-seq. Perhaps this results from the dual report of transcription and pause by PRO-seq. Alternatively, the small set of positive enhancers measured by reporter assays could result in having insufficient statistical power. Nevertheless, despite all these caveats, ATAC-seq and PRO-seq alone suffice to explain between one quarter and one fifth of the reporter enhancer activity in 20 h embryos (Fig. 5 and Fig. S5). It is reasonable to expect even better performance in single cell assays exclusively testing the genomic regions highlighted by ATAC- and PRO-seq profiles.

Our results confirm and extend reports of distinct enhancer prediction performance for promoter-proximal regulatory elements previously obtained with a distinct set of functional genomic profiles [11].

The higher enhancer activity predictive power of Pol II for the promoter-overlapping subset (Fig. 4G and Fig. S4 B and D) is not surprising, because the endogenous Pol II signal due to effective transcription should parallel transcription driven by the promoter of the reporter construct whenever relevant regulatory elements are included in the CRM (Fig. S3 A). Interestingly, ATAC and PRO-seq profiles are irrelevant for the prediction of promoter-overlapping CRMs in 20 h embryos (Fig. 4G and Fig. S4 C and D). The lower predictive power of ATAC and PRO-seq for the promoter overlapping subset may relate to enhancer sharing between the endogenous promoter and the reporter promoter (Fig. S3 A), which should be biased against the reporter promoter due to enhancer-promoter specificity. In other words, the endogenous promoter represents a perfect match and should more effectively sequester any local enhancers included in the CRM, therefore lowering reporter activity. This potentially confounding factor for promoters that are tested for enhancer activity in reporter assays may be difficult to address because the core promoters of enhancers are functional components required for their enhancer activity [18]. More interestingly, different from the 20 h embryos case, ATAC- and PRO-seq profiles have similar predictive power for promoter-overlapping CRMs relative to distal CRMs in 12 h embryos (Fig. S4 C and A). This suggests that distinct transcriptional regulatory mechanisms, including those related to enhancer-promoter specificity, may prevail at TREs used at different levels of the gene regulatory hierarchy that is sequentially deployed during sea urchin development [45], or that regulatory genes are at distinct stages in the sequence of events leading to their transcriptional activation during the 12 to 20 h transition. Alternatively, the potential enhancer-sharing bias against promoter-overlapping CRMs mentioned above may be more pronounced in the 20 h stage, when most of the 37 regulatory genes subject of this study [32] are activated (Fig. S6).

Along with major transcriptome changes during the 12 to 20 h transition [36], the majority of the 37 regulatory genes undergo more than a twofold change in transcript levels (Fig. S6), in agreement with their position in the topological models of the BioTapestry Interactive Network Viewer [45]. In short, the activation of the upstream regulatory genes that determine the main territorial subdivisions is underway in the 12 h blastula, while the transcriptional states determining these subdivisions are well stablished in the 20 h blastula, including the activation of terminal differentiation gene batteries at the periphery of the transcriptional network [21–23, 45, 52]. In general, more PRO-seq than ATAC-seq associated changes are observed during the 12 to 24 h transition (Fig. S1 F), in agreement with punctual observations (Fig. 3 and Fig. S2). This would follow the general sequence of events in the transcriptional cycle [53], with enhancer accessibility preceding transcriptional initiation, followed by pause and release, all of which are target of regulation by sequence specific transcription factors. The early territorial subdivisions are mediated both by transcriptional enhancer and silencer functions, and the relation of functional genomic profiles to experimental characterizations (Fig. 3 and Fig. S2) suggest that pausing may not only provide a venue for coordinated and prompt transcriptional activation during development [54], but also anticipate permanent silencing in some territories.

## Conclusions

In summary, ATAC- and PRO-seq are efficient predictors of reporter enhancer activity of distal CRMs in sea urchin embryos, while the prediction of promoter-overlapping CRMs is stage-dependent. In late blastula embryos, Pol II enrichment is the best predictor of promoter-proximal CRM enhancer activity. There is a net increase in dREG TRE predictions during later embryonic stages, while accessibility peaks remain relatively constant. In combination, this suggests that the sequence of regulatory events leading to developmental TRE enhancer activity has different relevance at different GRN levels or developmental stages. Our work facilitates ongoing developmental gene regulatory studies by mapping genome-wide candidate TREs, identifies PRO-seq and ATAC-seq as candidate factor-independent methods that predict developmental enhancer activity in whole embryos, and outlines the stage-dependency and predictive value of distinct functional genomic profiles associated with proximal and distal regulatory elements.

## Methods

### Preparation of nuclear extracts and sequencing libraries

Sea urchin embryos were reared to different stages as previously described [25]. Nuclei for ATAC-seq and PRO-seq were prepared using a modified version of a density gradient method [55] as follows. Sea urchin embryos were centrifuged at 500 g for 3 min at 0 °C, the pellet was resuspended in 10 volumes of ice cold lysis buffer consisting of 20 mM EDTA, 2% polyethylene glycol, and 4 mg/ml of Protease Inhibitor Tablets (Thermo Scientific™ Pierce™ #A32965), added just before use, in 0.1 X PBS (PBS is 0.137 mM NaCl, 2.7 mM Cl, 10 mM Na_2_HPO_4_ and 18 mM KH_2_PO_4_), and incubated on ice for 5 min. Dissociated cells were further disrupted with 50 or more strokes in a fine dounce homogenizer. Density gradient nuclear wash and floating layers were prepared by diluting iodixanol 60% (OptiPrep™) in 1 X PBS to 20 and 40%, respectively. About 5 ml of nuclear lysate was deposited on top of 10 ml of nuclear wash and nuclei were collected over 200 μl of floating layer after centrifugation for 30 min at 2 °C and 3000 g in a swing bucket rotor. Nuclei aliquots were flash-frozen in liquid nitrogen. For the 20 h stage, nuclei of one of the two ATAC-seq biological replicates was prepared as previously described [25]. For ChIP-seq, fixation was performed by resuspending embryo pellets in crosslinking solution (1 mM EDTA, 0.5 mM EGTA, 100 mM NaCl, 1.8% formaldehyde, 50 mM HEPES, pH 8.0) for 15 min at 22 °C, followed by gravity settling and subsequent resuspension in stop solution (125 mM glycine, 0.1% Triton X-100 in PBS), 500 g centrifugation, and two washes with PBT (0.1% Triton X-100 in PBS). The embryos were transferred to 25 ml of ice cold homogenization buffer (15 mM Tris-HCl pH 7.4, 0.34 M sucrose, 15 mM NaCl, 60 mM KCl, 0.2 mM EDTA, 0.2 mM EGTA, with 4 mg/ml protease inhibitors) and incubated on ice for 5 min. Embryos were first dounced 20 times with pestle type A (loose), followed by 10 times with pestle type B (tight). Nuclei were then filtered through a 20 μM filter and pelleted at 3500 g for 5 min at 4 °C. The nuclei were resuspended in 7.5 ml of PBTB (5% BSA in PBT buffer, with proteinase inhibitors). Propidium iodine stained nuclei were quantified using a hemocytometer and a fluorescence microscope.

ATAC-seq library preparation and Illumina sequencing followed similar procedures to those previously described [34]. The ENCOCE-DCC atac-seq-pipeline [56] was used for mapping the raw reads to the *S. purpuratus* genome version 3.1 using default settings, except for the MACS2 peak call p threshold, which was set to 0.05. Two biological and one technical replicate libraries were paired or single end sequenced. A total of 16,005,927 20-h and 73,272,494 12-h embryo reads mapped to the genome after deduplication and mitochondrial chromosome filtration. About 44% of the reads locate in peak calls, see quality control summary for detailed reproducibility and sequence quality metrics (Supplementary quality control files).

PROseq libraries were elaborated following previously stablished protocols [17], single or paired end sequenced in Illumina platforms, and mapped to the *S. purpuratus* genome version 3.1 using proseq2.0 pipeline [57]. Regulatory elements were predicted with the vector machine learning tool dREG [15]. Two biological and one technical replicate libraries were prepared and paired or single end sequenced, providing a total of 56,781,051 20-h and 28,430,031 12-h mapped reads, excluding ribosomal RNAs.

For ChIP-seq library preparation, 50 to 100 million nuclei were spun at 4000 g for 5 min at 4 °C. Nuclei were resuspended in 1 ml FA buffer (50 mM HEPES/KPH pH 7.5, 1 mM EDTA, 1% Triton X-100, 0.1% sodium deoxycholate, 150 mM NaCl, 0.1% sarkosyl, and protease and phosphatase inhibitors). The resuspended nuclei were then sonicated at 4 °C for 15 min on high, cycle of 30 s on and 30 s off, to obtain an extract with fragmented chromatin. Extracts were brought up to 440 μL with FA buffer with protease and phosphatase inhibitors. Between 1 to 2 mg of extract and 4 μg of antibody were used per ChIP. Prior to the addition of antibody, 5% of the extract was taken for input. Mouse monoclonal antibody against RNA polymerase II CTD-repeat YSPTSPS (8WG16; abcam ab817mod) was used for ChIP. The mixture was incubated rotating at 4 °C overnight. 40 μL of protein G sepharose bead slurry (GE Healthcare) per ChIP sample was washed three times with 1 mL FA buffer, added 40 μL bead slurry to each ChIP sample and rotated at 4 °C for 2 h. Meanwhile, 200 μL ChIP Elution Buffer (1% SDS, 250 mM NaCl, 10 mM Tris pH 8.0, 1 mM EDTA) and 2 μL 10 mg/μL RNase A were added to inputs and incubated at room temperature. Beads were washed at room temperature by adding 1 mL of each of the following buffers and collecting beads by spinning for 1 min at 2500 g: two times FA buffer for 5 min, one time FA-1 M NaCl for 5 min, one time FA-500 mM NaCl for 10 min, one time TEL buffer (0.25 M LiCl, 1% NP-40, 1% sodium deoxycholate, 1 mM EDTA, 10 mM Tris-HCl, pH 8.0) for 10 min, two times TE for 5 min. Proteinase K was added to both inputs and ChIPs and incubated in a 50 °C heat block for an hour. Inputs and ChIPs were allowed to reverse crosslink overnight in a 65 °C water bath. DNA was ligated to Illumina or homemade multiplexed adapters and amplified by PCR. Using a thin 1.5% agarose gel, DNA fragments between 300 and 600 bp were purified using the Qiagen Gel Extraction kit. Qubit flourometer was used to measure DNA concentration. Single-end sequencing was performed for the ChIP-seq and input DNA at the New York University Center for Genomics and Systems Biology high-throughput sequencing facility. We combined replicates and aligned 50 bp single end reads to the *S. purpuratus* genome version 3.1 linear scaffolds using Bowtie 2 version 2.2.3 [58] with default parameters. A total of 13,762,893 ChIP and 26,476,643 input mapped reads were obtained. Mapped reads from ChIP and input were used to call peaks and coverage per base using MACS version 1.4.2 [59] with default parameters.

### Computational analysis of PRO-, ChIP-, and ATAC-seq peak calls and machine learning, enhancer activity prediction

Signal at PRO-, ChIP-, and ATAC-seq peak calls was quantified using the R package bigWig [60]. ATAC and PRO-seq reads of 12 and 20 h embryos where normalized to reads per million per base. For ATAC-seq peak calls, any overlapping peaks were merged prior to analysis. Density plots used R lift posted in StackOverflow [61]. Overlaps among PRO-, ChIP-, and ATAC-seq peak calls were analyzed and illustrated with ChIPpeakAnno [62] using default parameters. Promoters are defined as the 200 bp region centered at the 5′ end of transcript based gene models, which are a better approximations than GLEAN models [36]. For all data sets, reads from different replicates were merged into single bigwig files and reads computed at peak calls and dREG predictions using the bigWig interface [60].

Using bedtools [63], the intersections between dREG predictions and CRMs were merged, to correct for CRM overlaps, and then extended 50 bp, to compute the pause associated PRO-seq reads oftentimes extending beyond the raw dREG prediction. The total number of 3′ end reads in the plus and minus strand and the summit for each TRE prediction was estimated with the sum and max parameters of the bigWig query function. Similar analysis was performed for the reads per base for the intersection with ATAC- and Pol II ChIP peaks, without the 50 bp extension. Graphics were elaborated with ggplot and tidyverse [64].

The package caret was used for the optimization, test and evaluation of classification and regression models [65]. Logistic classification and linear regression models were fitted and tested by 5 fold cross-validation with stratified sampling repeated 200 times. The package precrec [66] was used to generate ROCs and PRCs.

## Supplementary Information

**Additional file 1.**

**Additional file 2: Supplementary Table 1.** Genomic locations, genomic signals and reporter activities of the 389 CRMs.

**Additional file 3: Fig. S1.** Promoter and CRM PRO-, ATAC- and ChIP-seq analysis.

**Additional file 4: Fig. S2.** PRO-, Pol II ChIP- and ATAC-seq at *onecut, gcm, gatae* and *delta*. A, onecut, the entire region was scanned for enhancer activity by overlapping reporter constructs averaging 2.23 Kb [40]. Similar snapshots are taken for other regulatory genes whose regulatory elements were primarily selected based on evolutionary sequence conservation rather than an unbiased tiling scan.

**Additional file 5: Fig. S3.** The enhancer activity of promoter-overlapping CRMs is independent of their orientation. A, the concurrent orientation of the promoter in the GFP reporter construct and the promoter of promoter-overlapping CRMs could in principle result in transcripts reaching to the coding region of the reporter that could confound CRM driven transcription (red dashed line) with enhancer activity (black dotted line). In both cases the reporter enhancer activity of CRMs containing endogenous promoters should be diminished due to enhancer sharing between both promoters. Correlation of Pol II accumulation at endogenous promoters (purple ovals) and reporter expression is expected for CRMs that contain necessary enhancers and endogenous promoters. B, the difference of expression between CRMs in concurrent and divergent reporter construct orientation is not significant (ns) in 12 and 24 h embryos, Wilcox test *p*-values of 0.67 and 0.14, respectively.

**Additional file 6: Fig. S4.** Evaluation of models for CRMs overlapping and not overlapping promoters. A, 12 h embryo and, B, 20 h embryo models trained and tested with CRMs not overlapping promoters. C, 12 h embryo and, D, 20 h embryo models trained and tested with CRMs overlapping promoters.

**Additional file 7: Fig. S5.** Quantitative prediction of enhancer activity from ATAC and PRO-seq data. Plot of the hold-out predicted and actual reporter expression of linear regression models using ATAC and PRO-seq signal in 12 h and 20 h stages. Violin/Box-plots of R^2^ values, with the average indicated underneath.

**Additional file 8: Fig. S6.** Summary 12 to 20 h mRNA expression changes of regulatory genes. The fold change in the mRNA expression levels quantified at high resolution for 31 of the 37 genes used in this study [33].

## Data Availability

The datasets generated and analyzed during the current study are available at NCBI GEO under accession number GSE160463 [44]. https://www.ncbi.nlm.nih.gov/geo/query/acc.cgi?acc=GSE160463

## Abbreviations

ATAC-seq: Assay for Transposase-Accessible Chromatin using sequencing
AUPRC: Area under the Precision-Recall Curve
AUROC: Area under the Receiver Operating Characteristic Curve
BAC: Bacterial Artificial Chromosome
ChIP-seq: Chromatin Immunoprecipitation followed by sequencing
CRM: *cis-*regulatory module
dREG: Discriminative regulatory-element detection from GRO-seq
GRN: Gene regulatory network
MACS: Model-based Analysis for ChIP-seq
Pol II: RNA polymerase II
PRC: Precision-Recall Curve
PRO-seq: Precision nuclear run-on sequencing
ROC: Receiver Operating Characteristic Curve
TRE: Transcriptional regulatory element
TSS: Transcription start site
