# Supplementary material for: Identification and prediction of developmental enhancers in sea urchin embryos
Source: BMC Genomics. 2021 Oct 19;22:751. doi: 10.1186/s12864-021-07936-0 (PMC8527612; doi:10.1186/s12864-021-07936-0)
Supplement: Supplementary file 1 — Additional file 1. [file 12864_2021_7936_MOESM1_ESM.pdf]

QC Report

	general
**Report generated at**	2020-10-23 01:02:22
**Title**	ATAC_12h_3.1
**Description**	PE 12h
**Pipeline version**	v1.7.0
**Pipeline type**	atac
**Genome**	Sp3.1
**Aligner**	bowtie2
**Sequencing endedness**	{'rep1': {'paired_end': True}, 'rep2': {'paired_end': True}}
**Peak caller**	macs2

Alignment quality metrics

SAMstat (raw unfiltered BAM)

	rep1	rep2
**Total Reads**	161870122	141132800
**Total Reads (QC-failed)**	0	0
**Duplicate Reads**	0	0
**Duplicate Reads (QC-failed)**	0	0
**Mapped Reads**	99587727	111643482
**Mapped Reads (QC-failed)**	0	0
**% Mapped Reads**	61.5	79.10000000000001
**Paired Reads**	161870122	141132800
**Paired Reads (QC-failed)**	0	0
**Read1**	80935061	70566400
**Read1 (QC-failed)**	0	0
**Read2**	80935061	70566400
**Read2 (QC-failed)**	0	0
**Properly Paired Reads**	98226484	109823926
**Properly Paired Reads (QC-failed)**	0	0
**% Properly Paired Reads**	60.699999999999996	77.8
**With itself**	98678724	110336158
**With itself (QC-failed)**	0	0
**Singletons**	909003	1307324
**Singletons (QC-failed)**	0	0
**% Singleton**	0.6	0.8999999999999999
**Diff. Chroms**	16547	27794
**Diff. Chroms (QC-failed)**	0	0

Marking duplicates (filtered BAM)

	rep1	rep2
**Unpaired Reads**	0	0
**Paired Reads**	20991029	23442940
**Unmapped Reads**	0	0
**Unpaired Duplicate Reads**	0	0
**Paired Duplicate Reads**	3281595	4516127
**Paired Optical Duplicate Reads**	118207	140189
**% Duplicate Reads**	15.6333	19.264300000000002

Filtered out (samtools view -F 1804):

- read unmapped (0x4)
- mate unmapped (0x8, for paired-end)
- not primary alignment (0x100)
- read fails platform/vendor quality checks (0x200)
- read is PCR or optical duplicate (0x400)

Fraction of mitochondrial reads (unfiltered BAM)

	rep1	rep2
**Rn = Number of Non-mitochondrial Reads**	99587727	111643482
**Rm = Number of Mitochondrial Reads**	33967040	3532090
**Rm/(Rn+Rm) = Frac. of mitochondrial reads**	0.2543304201189614	0.03066700636832956

SAMstat (filtered/deduped BAM)

	rep1	rep2
**Total Reads**	35418868	37853626
**Total Reads (QC-failed)**	0	0
**Duplicate Reads**	0	0
**Duplicate Reads (QC-failed)**	0	0
**Mapped Reads**	35418868	37853626
**Mapped Reads (QC-failed)**	0	0
**% Mapped Reads**	100.0	100.0
**Paired Reads**	35418868	37853626
**Paired Reads (QC-failed)**	0	0
**Read1**	17709434	18926813
**Read1 (QC-failed)**	0	0
**Read2**	17709434	18926813
**Read2 (QC-failed)**	0	0
**Properly Paired Reads**	35418868	37853626
**Properly Paired Reads (QC-failed)**	0	0

**% Properly Paired Reads**	100.0	100.0
**With itself**	35418868	37853626
**With itself (QC-failed)**	0	0
**Singletons**	0	0
**Singletons (QC-failed)**	0	0
**% Singleton**	0.0	0.0
**Diff. Chroms**	0	0
**Diff. Chroms (QC-failed)**	0	0

Filtered and duplicates removed

Fragment length statistics (filtered/deduped BAM)

	rep1	rep2
**Fraction of reads in NFR**	0.9323787083560022	0.8902688074183567
**Fraction of reads in NFR (QC pass)**	True	True
**Fraction of reads in NFR (QC reason)**	OK	OK
**NFR / mono-nuc reads**	15.598049844737124	9.061774693997215
**NFR / mono-nuc reads (QC pass)**	True	True
**NFR / mono-nuc reads (QC reason)**	OK	OK
**Presence of NFR peak**	True	True
**Presence of Mono-Nuc peak**	False	True
**Presence of Di-Nuc peak**	False	False

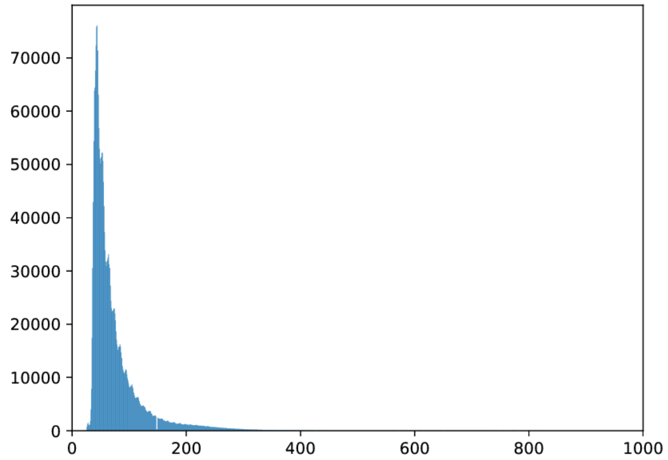

rep1

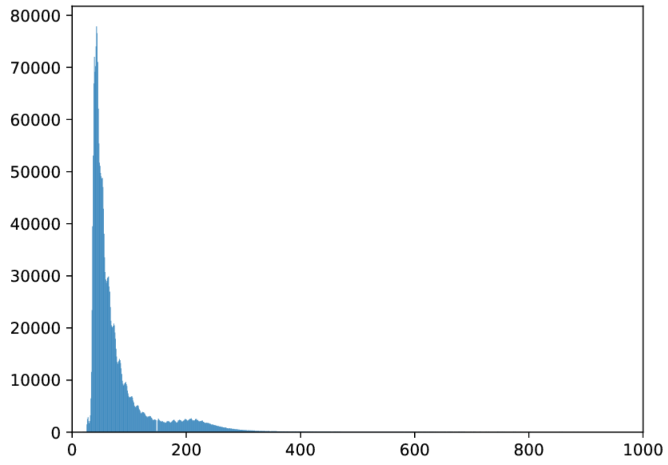

rep2

Open chromatin assays show distinct fragment length enrichments, as the cut sites are only in open chromatin and not in nucleosomes. As such, peaks representing different n-nucleosomal (ex mono-nucleosomal, di-nucleosomal) fragment lengths will arise. Good libraries will show these peaks in a fragment length distribution and will show specific peak ratios.

- NFR: Nucleosome free region

Sequence quality metrics (filtered/deduped BAM)

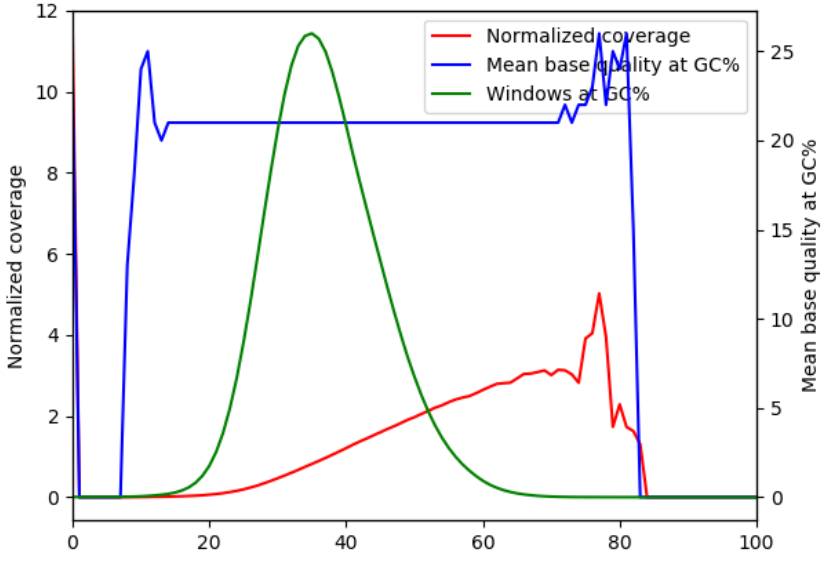

rep1

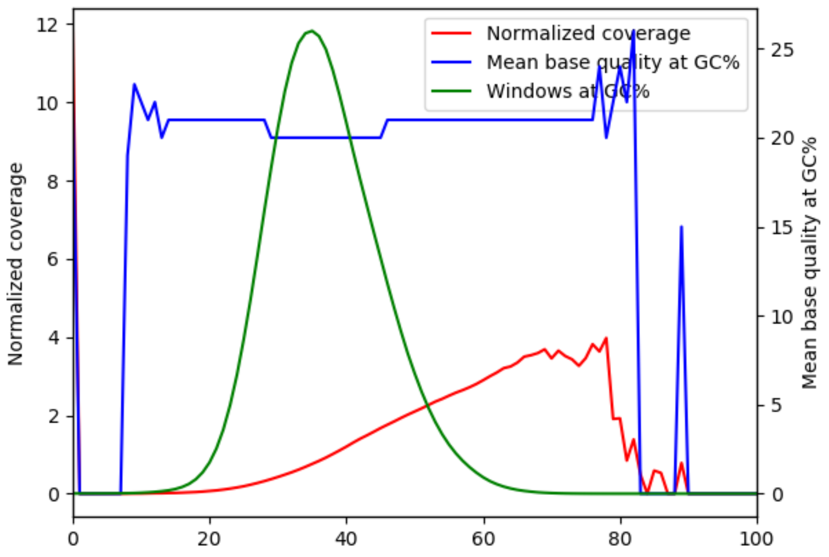

rep2

Open chromatin assays are known to have significant GC bias. Please take this into consideration as necessary.

Library complexity quality metrics

Library complexity (filtered non-mito BAM)

	rep1	rep2
**Total Fragments**	20991029	23442940
**Distinct Fragments**	17920070	19255358
**Positions with Two Read**	2196429	2734948
**NRF = Distinct/Total**	0.853701	0.821371
**PBC1 = OneRead/Distinct**	0.856182	0.825603
**PBC2 = OneRead/TwoRead**	6.985358	5.812643

Mitochondrial reads are filtered out by default. The non-redundant fraction (NRF) is the fraction of non-redundant mapped reads in a dataset; it is the ratio between the number of positions in the genome that uniquely mapped reads map to and the total number of uniquely mappable reads. The NRF should be > 0.8. The PBC1 is the ratio of genomic locations with EXACTLY one read pair over the genomic locations with AT LEAST one read pair. PBC1 is the primary measure, and the PBC1 should be close to 1. Provisionally 0-0.5 is severe bottlenecking, 0.5-0.8 is moderate bottlenecking, 0.8-0.9 is mild bottlenecking, and 0.9-1.0 is no bottlenecking. The PBC2 is the ratio of genomic locations with EXACTLY one read pair over the genomic locations with EXACTLY two read pairs. The PBC2 should be significantly greater than 1.

NRF (non redundant fraction)
PBC1 (PCR Bottleneck coefficient 1)
PBC2 (PCR Bottleneck coefficient 2)
PBC1 is the primary measure. Provisionally

- 0-0.5 is severe bottlenecking
- 0.5-0.8 is moderate bottlenecking
- 0.8-0.9 is mild bottlenecking
- 0.9-1.0 is no bottlenecking

Replication quality metrics

IDR (Irreproducible Discovery Rate) plots

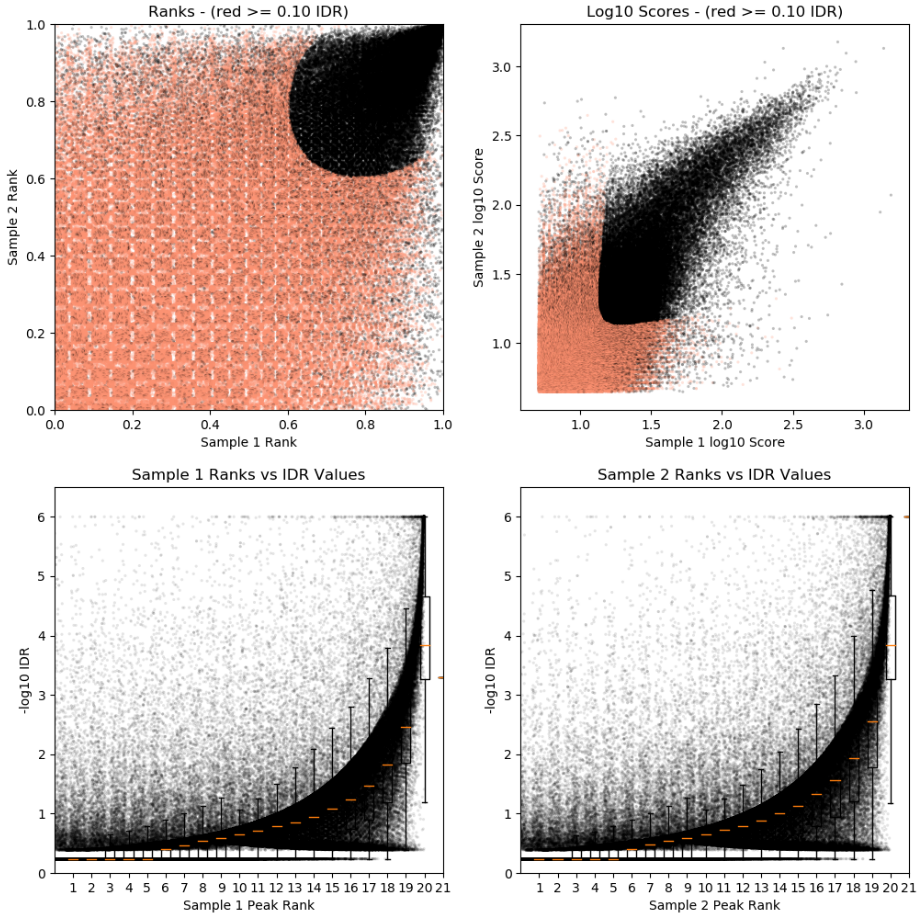

rep1_vs_rep2

Number of raw peaks

	rep1	rep2
**Number of peaks**	300000	300000

Top 300000 raw peaks from macs2 with p-val threshold 0.05

Peak calling statistics

Peak region size

	rep1	rep2
**Min size**	150.0	150.0
**25 percentile**	223.0	223.0
**50 percentile (median)**	348.0	355.0

**75 percentile**	567.0	582.0
**Max size**	3602.0	4282.0
**Mean**	453.15470666666664	461.8133433333333

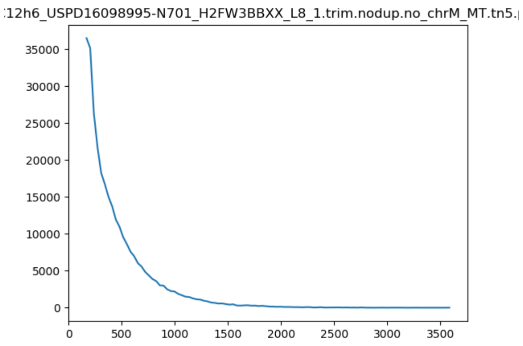

rep1

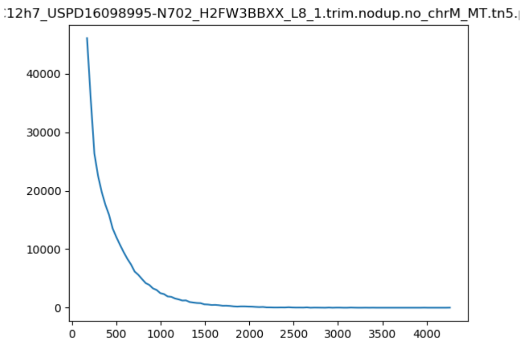

rep2

Peak enrichment

Fraction of reads in peaks (FRiP)

FRiP for macs2 raw peaks

	rep1	rep2	pooled
**Fraction of Reads in Peaks**	0.5425538444650462	0.5892748821473536	0.5670562817201227

FRiP for overlap peaks

	rep1_vs_rep2
**Fraction of Reads in Peaks**	0.4852075732538871

FRiP for IDR peaks

	rep1_vs_rep2
**Fraction of Reads in Peaks**	0.2598057328306581

For macs2 raw peaks:

- repX: Peak from true replicate X
- repX-prY: Peak from Yth pseudoreplicates from replicate X
- pooled: Peak from pooled true replicates (pool of rep1, rep2, ...)
- pooled-pr1: Peak from 1st pooled pseudo replicate (pool of rep1-pr1, rep2-pr1, ...)
- pooled-pr2: Peak from 2nd pooled pseudo replicate (pool of rep1-pr2, rep2-pr2, ...)

For overlap/IDR peaks:

- repX_vs_repY: Comparing two peaks from true replicates X and Y
- repX-pr1_vs_repX-pr2: Comparing two peaks from both pseudoreplicates from replicate X
- pooled-pr1_vs_pooled-pr2: Comparing two peaks from 1st and 2nd pooled pseudo replicates

QC Report

	general
**Report generated at**	2020-04-22 02:18:15
**Title**	ATAC_20h_3.1
**Description**	PE and SR
**Pipeline version**	v1.7.0
**Pipeline type**	atac
**Genome**	Sp3.1
**Aligner**	bowtie2
**Sequencing endedness**	{'rep1': {'paired_end': True}, 'rep2': {'paired_end': False}, 'rep3': {'paired_end': False}}
**Peak caller**	macs2

Alignment quality metrics

SAMstat (raw unfiltered BAM)

	rep1	rep2	rep3
**Total Reads**	20150554	71283893	8095974
**Total Reads (QC-failed)**	0	0	0
**Duplicate Reads**	0	0	0
**Duplicate Reads (QC-failed)**	0	0	0
**Mapped Reads**	15124852	54426636	5482360
**Mapped Reads (QC-failed)**	0	0	0
**% Mapped Reads**	75.1	76.4	67.7
**Paired Reads**	20150554	0	0
**Paired Reads (QC-failed)**	0	0	0
**Read1**	10075277	0	0
**Read1 (QC-failed)**	0	0	0
**Read2**	10075277	0	0
**Read2 (QC-failed)**	0	0	0
**Properly Paired Reads**	13712336	0	0
**Properly Paired Reads (QC-failed)**	0	0	0
**% Properly Paired Reads**	68.0	0.0	0.0
**With itself**	14349280	0	0
**With itself (QC-failed)**	0	0	0
**Singletons**	775572	0	0
**Singletons (QC-failed)**	0	0	0
**% Singleton**	3.8	0.0	0.0
**Diff. Chroms**	136751	0	0
**Diff. Chroms (QC-failed)**	0	0	0

Marking duplicates (filtered BAM)

	rep1	rep2	rep3
**Unpaired Reads**	0	22783394	2498208
**Paired Reads**	2994364	0	0
**Unmapped Reads**	0	0	0
**Unpaired Duplicate Reads**	0	14484938	195021
**Paired Duplicate Reads**	292222	0	0
**Paired Optical Duplicate Reads**	295	0	0
**% Duplicate Reads**	9.7591	63.576699999999995	7.806399999999999

Filtered out (samtools view -F 1804):

- read unmapped (0x4)
- mate unmapped (0x8, for paired-end)
- not primary alignment (0x100)
- read fails platform/vendor quality checks (0x200)
- read is PCR or optical duplicate (0x400)

Fraction of mitochondrial reads (unfiltered BAM)

	rep1	rep2	rep3
**Rn = Number of Non-mitochondrial Reads**	15124852	54426636	5482360
**Rm = Number of Mitochondrial Reads**	153339	589223	1311787
**Rm/(Rn+Rm) = Frac. of mitochondrial reads**	0.010036463086500228	0.010710057258217127	0.1930760403035142

SAMstat (filtered/deduped BAM)

	rep1	rep2	rep3
**Total Reads**	5404284	8298456	2303187
**Total Reads (QC-failed)**	0	0	0
**Duplicate Reads**	0	0	0
**Duplicate Reads (QC-failed)**	0	0	0
**Mapped Reads**	5404284	8298456	2303187
**Mapped Reads (QC-failed)**	0	0	0
**% Mapped Reads**	100.0	100.0	100.0
**Paired Reads**	5404284	0	0
**Paired Reads (QC-failed)**	0	0	0
**Read1**	2702142	0	0
**Read1 (QC-failed)**	0	0	0
**Read2**	2702142	0	0
**Read2 (QC-failed)**	0	0	0

**Properly Paired Reads**	5404284	0	0
**Properly Paired Reads (QC-failed)**	0	0	0
**% Properly Paired Reads**	100.0	0.0	0.0
**With itself**	5404284	0	0
**With itself (QC-failed)**	0	0	0
**Singletons**	0	0	0
**Singletons (QC-failed)**	0	0	0
**% Singleton**	0.0	0.0	0.0
**Diff. Chroms**	0	0	0
**Diff. Chroms (QC-failed)**	0	0	0

Filtered and duplicates removed

Fragment length statistics (filtered/deduped BAM)

	**rep1**
**Fraction of reads in NFR**	0.6477683030192886
**Fraction of reads in NFR (QC pass)**	True
**Fraction of reads in NFR (QC reason)**	OK
**NFR / mono-nuc reads**	2.733468616968029
**NFR / mono-nuc reads (QC pass)**	True
**NFR / mono-nuc reads (QC reason)**	OK
**Presence of NFR peak**	True
**Presence of Mono-Nuc peak**	True
**Presence of Di-Nuc peak**	True

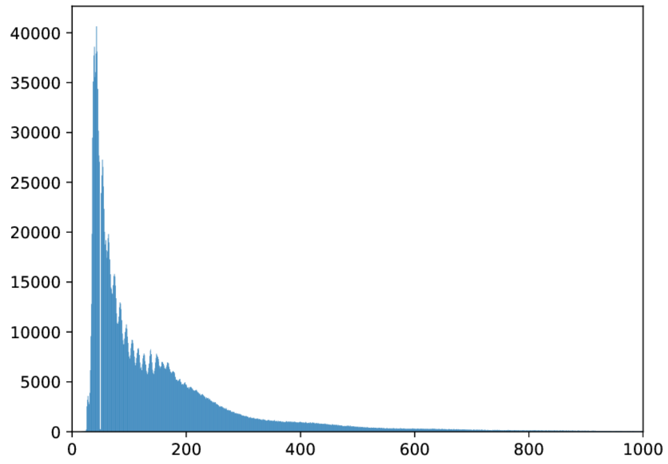

rep1

Open chromatin assays show distinct fragment length enrichments, as the cut sites are only in open chromatin and not in nucleosomes. As such, peaks representing different n-nucleosomal (ex mono-nucleosomal, di-

nucleosomal) fragment lengths will arise. Good libraries will show these peaks in a fragment length distribution and will show specific peak ratios.

- NFR: Nucleosome free region

Sequence quality metrics (filtered/deduped BAM)

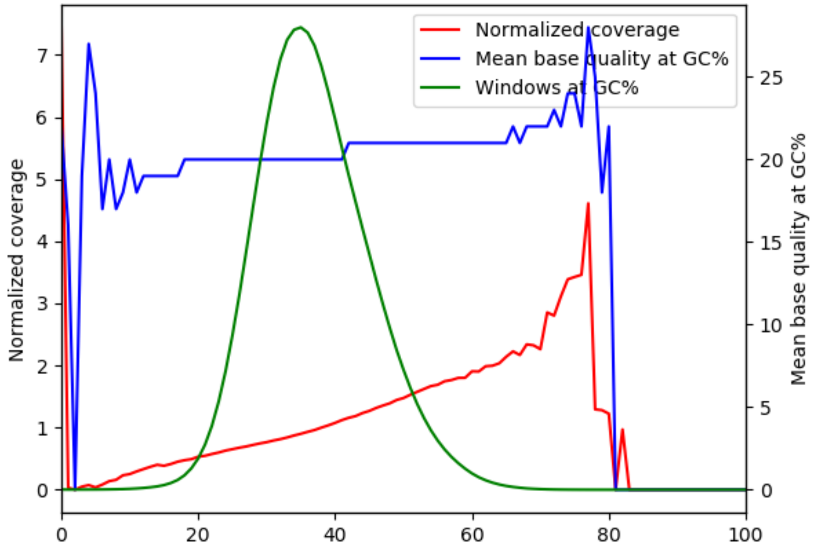

rep1

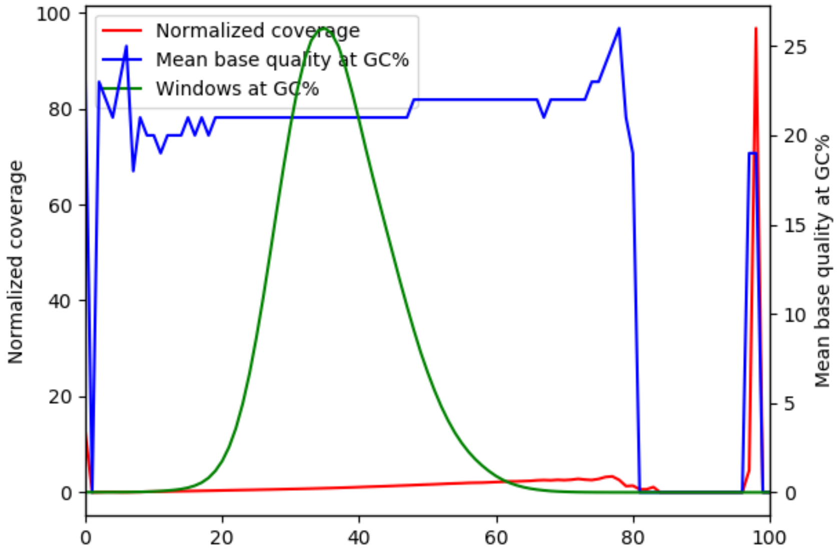

rep2

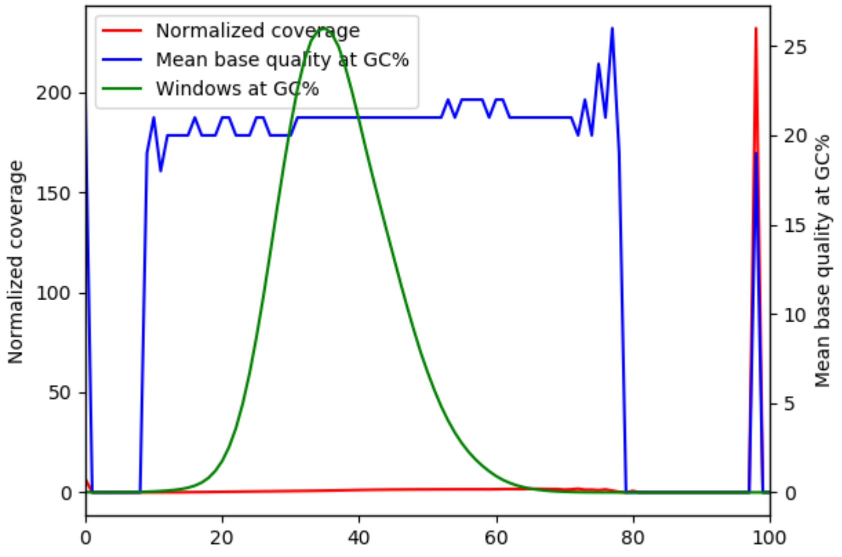

rep3

Open chromatin assays are known to have significant GC bias. Please take this into consideration as necessary.

Library complexity quality metrics

Library complexity (filtered non-mito BAM)

	rep1	rep2	rep3
**Total Fragments**	2994364	22783394	2498208
**Distinct Fragments**	2702613	8533758	2335470
**Positions with Two Read**	243447	2119342	119191
**NRF = Distinct/Total**	0.902567	0.37456	0.934858
**PBC1 = OneRead/Distinct**	0.901346	0.318471	0.941364
**PBC2 = OneRead/TwoRead**	10.00624	1.282357	18.44542

Mitochondrial reads are filtered out by default. The non-redundant fraction (NRF) is the fraction of non-redundant mapped reads in a dataset; it is the ratio between the number of positions in the genome that uniquely mapped reads map to and the total number of uniquely mappable reads. The NRF should be > 0.8. The PBC1 is the ratio of genomic locations with EXACTLY one read pair over the genomic locations with AT LEAST one read pair. PBC1 is the primary measure, and the PBC1 should be close to 1. Provisionally 0-0.5 is severe bottlenecking, 0.5-0.8 is moderate bottlenecking, 0.8-0.9 is mild bottlenecking, and 0.9-1.0 is no bottlenecking. The PBC2 is the ratio of genomic locations with EXACTLY one read pair over the genomic locations with EXACTLY two read pairs. The PBC2 should be significantly greater than 1.

NRF (non redundant fraction)
PBC1 (PCR Bottleneck coefficient 1)
PBC2 (PCR Bottleneck coefficient 2)
PBC1 is the primary measure. Provisionally

- 0-0.5 is severe bottlenecking
- 0.5-0.8 is moderate bottlenecking
- 0.8-0.9 is mild bottlenecking
- 0.9-1.0 is no bottlenecking

Replication quality metrics

IDR (Irreproducible Discovery Rate) plots

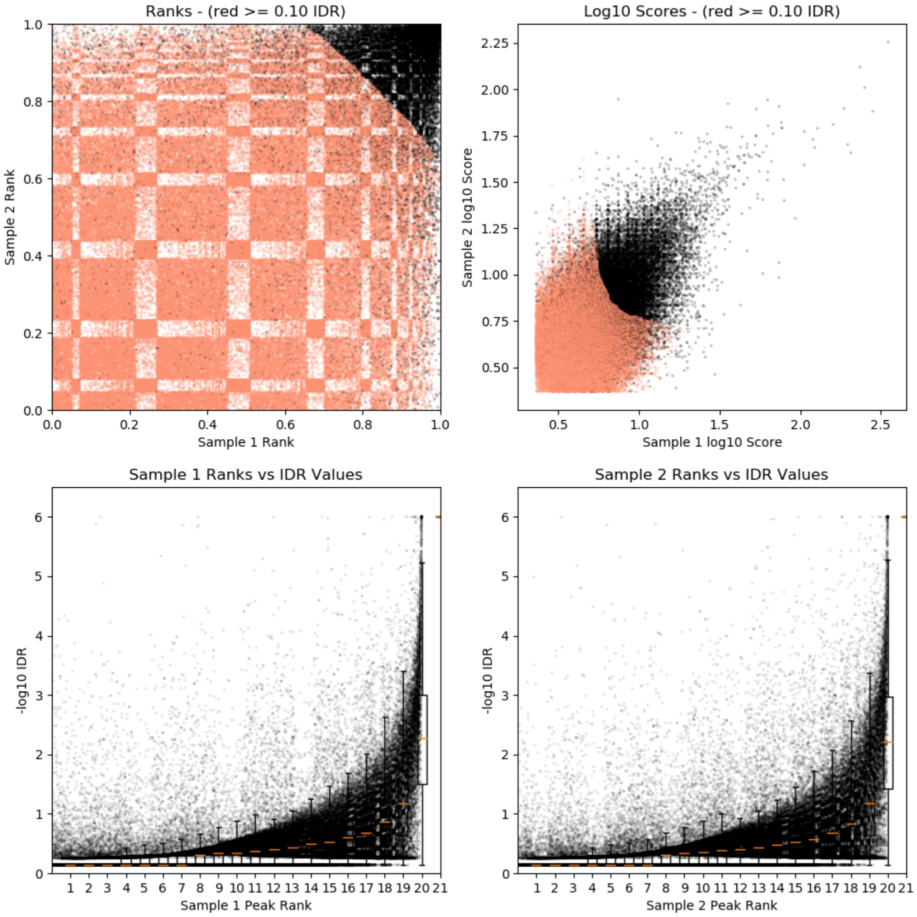
Ranks - (red >= 0.10 IDR)
Sample 2 Rank
Sample 1 Rank
Log10 Scores - (red >= 0.10 IDR)
Sample 2 log10 Score
Sample 1 log10 Score
Sample 1 Ranks vs IDR Values
-log10 IDR
Sample 1 Peak Rank
Sample 2 Ranks vs IDR Values
-log10 IDR
Sample 2 Peak Rank

rep1_vs_rep2

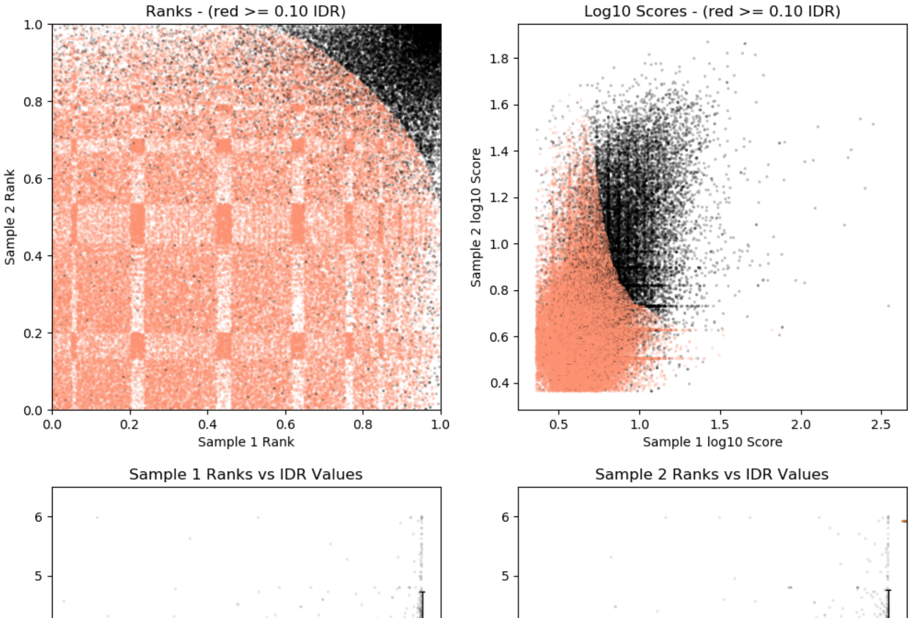
Ranks - (red >= 0.10 IDR)
Sample 2 Rank
Sample 1 Rank
Log10 Scores - (red >= 0.10 IDR)
Sample 2 log10 Score
Sample 1 log10 Score
Sample 1 Ranks vs IDR Values
Sample 2 Ranks vs IDR Values

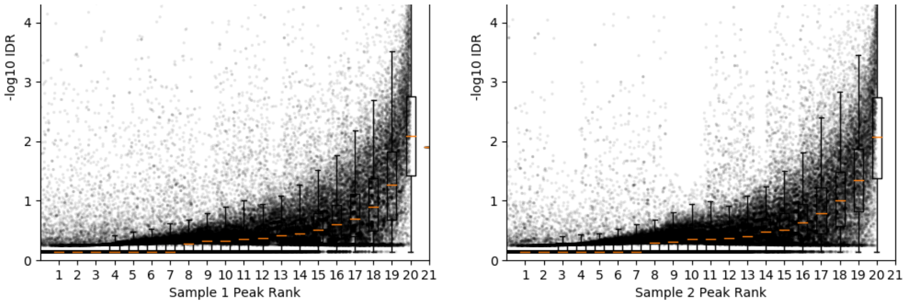

rep1_vs_rep3

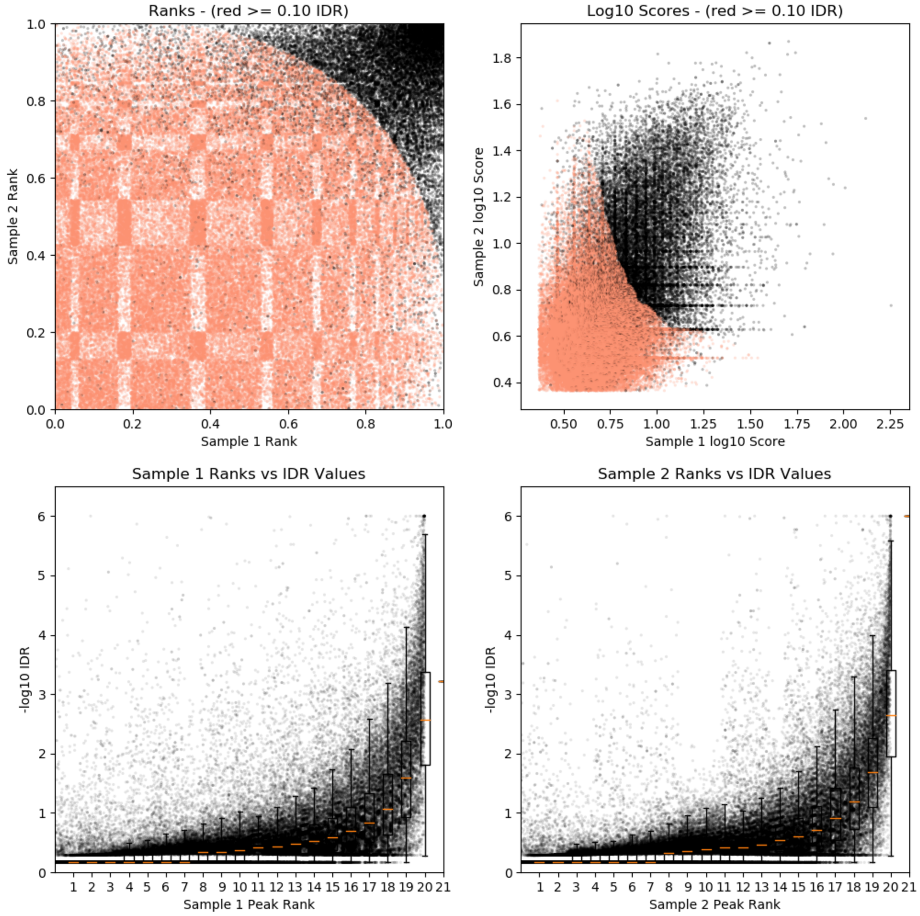

rep2_vs_rep3

Number of raw peaks

	rep1	rep2	rep3
**Number of peaks**	248700	300000	160654

Top 300000 raw peaks from macs2 with p-val threshold 0.05

Peak calling statistics

Peak region size

	rep1	rep2	rep3
**Min size**	150.0	150.0	150.0
**25 percentile**	157.0	166.0	150.0
**50 percentile (median)**	212.0	230.0	182.0
**75 percentile**	313.0	353.0	299.0
**Max size**	3115.0	4044.0	2008.0
**Mean**	270.7728266988339	302.13554666666664	266.0243317937929

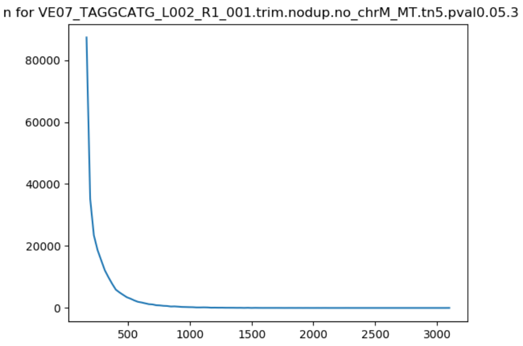

rep1

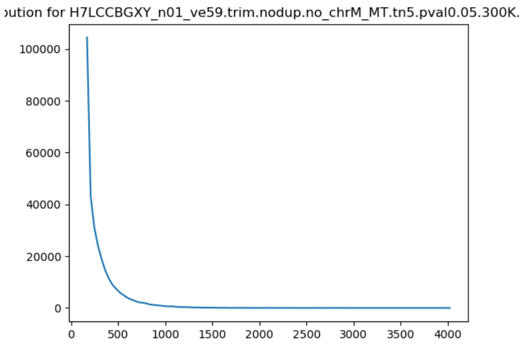

rep2

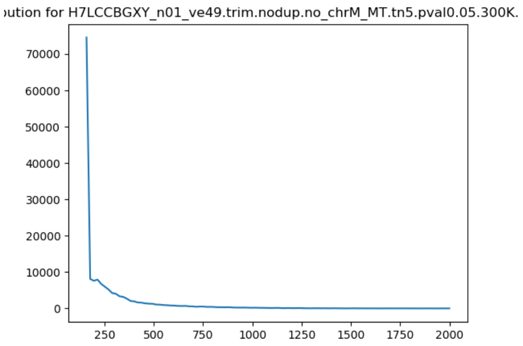

rep3

Peak enrichment

Fraction of reads in peaks (FRiP)

FRiP for macs2 raw peaks

	rep1	rep2	rep3	pooled
**Fraction of Reads in Peaks**	0.4482899122251902	0.44582546439964255	0.495024937184866	0.44154006200328166

FRiP for overlap peaks

	rep1_vs_rep2	rep1_vs_rep3	rep2_vs_rep3
**Fraction of Reads in Peaks**	0.29597523467400544	0.1967762317046679	0.21487827602862364

FRiP for IDR peaks

	rep1_vs_rep2	rep1_vs_rep3	rep2_vs_rep3
**Fraction of Reads in Peaks**	0.07651753003746675	0.05970275885926507	0.07640663361765926

For macs2 raw peaks:

- repX: Peak from true replicate X
- repX-prY: Peak from Yth pseudoreplicates from replicate X
- pooled: Peak from pooled true replicates (pool of rep1, rep2, ...)
- pooled-pr1: Peak from 1st pooled pseudo replicate (pool of rep1-pr1, rep2-pr1, ...)
- pooled-pr2: Peak from 2nd pooled pseudo replicate (pool of rep1-pr2, rep2-pr2, ...)

For overlap/IDR peaks:

- repX_vs_repY: Comparing two peaks from true replicates X and Y
- repX-pr1_vs_repX-pr2: Comparing two peaks from both pseudoreplicates from replicate X
- pooled-pr1_vs_pooled-pr2: Comparing two peaks from 1st and 2nd pooled pseudo replicates
