# Supplementary figures and images for: Identification and prediction of developmental enhancers in sea urchin embryos
Source: BMC Genomics. 2021 Oct 19;22:751. doi: 10.1186/s12864-021-07936-0 (PMC8527612; doi:10.1186/s12864-021-07936-0)

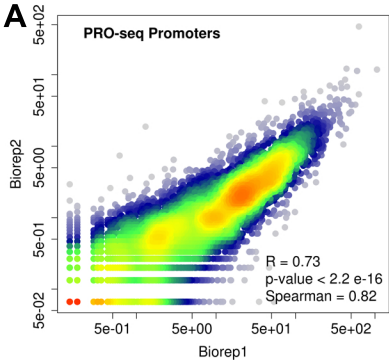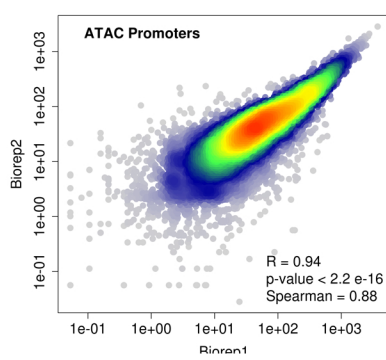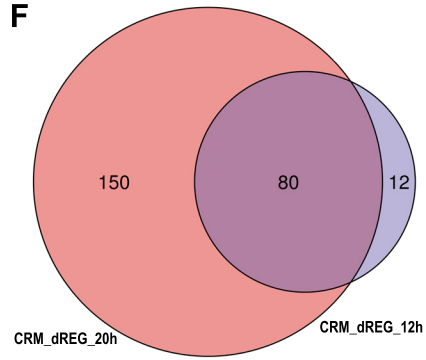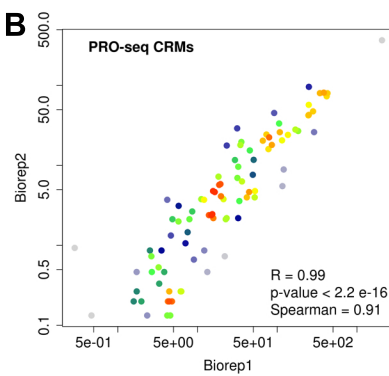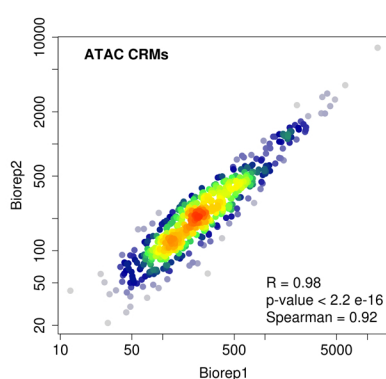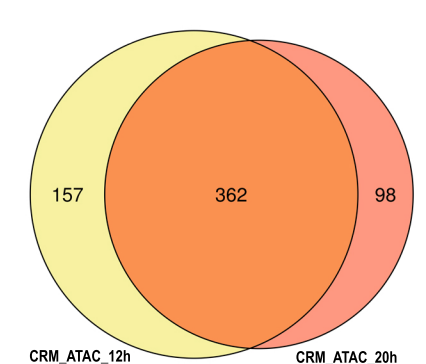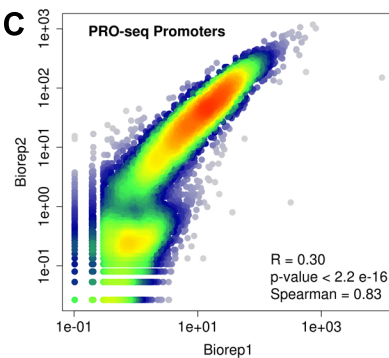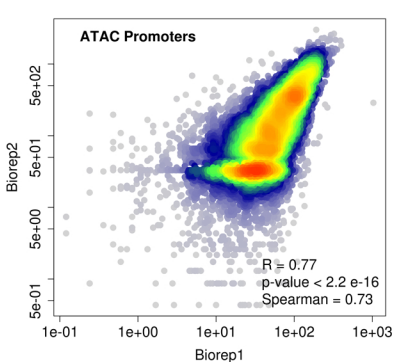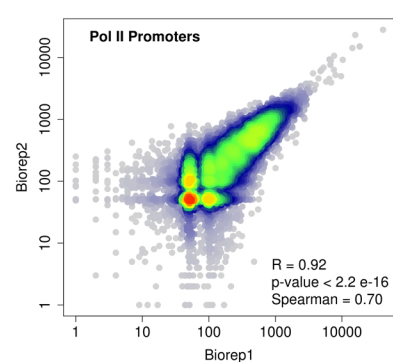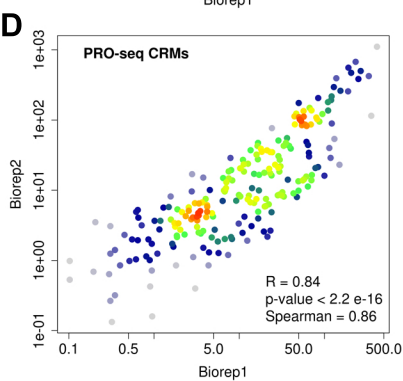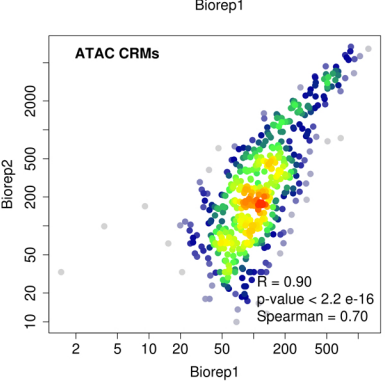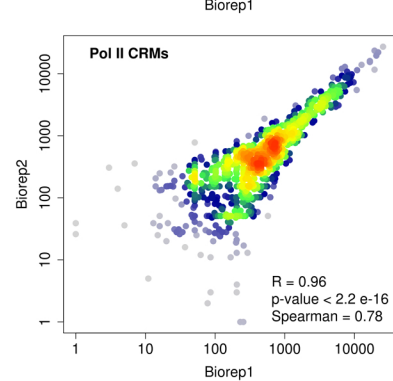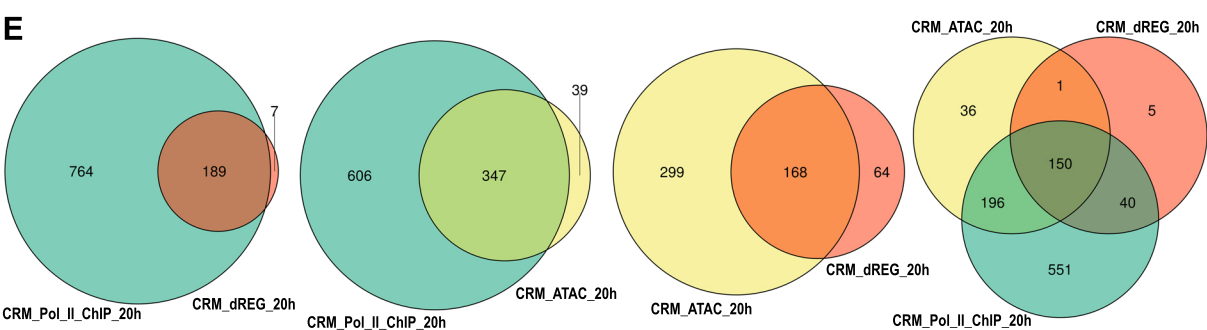

Supplement: Supplementary file 3 — Additional file 3: Fig. S1. Promoter and CRM PRO-, ATAC- and ChIP-seq analysis. [file 12864_2021_7936_MOESM3_ESM.pdf]

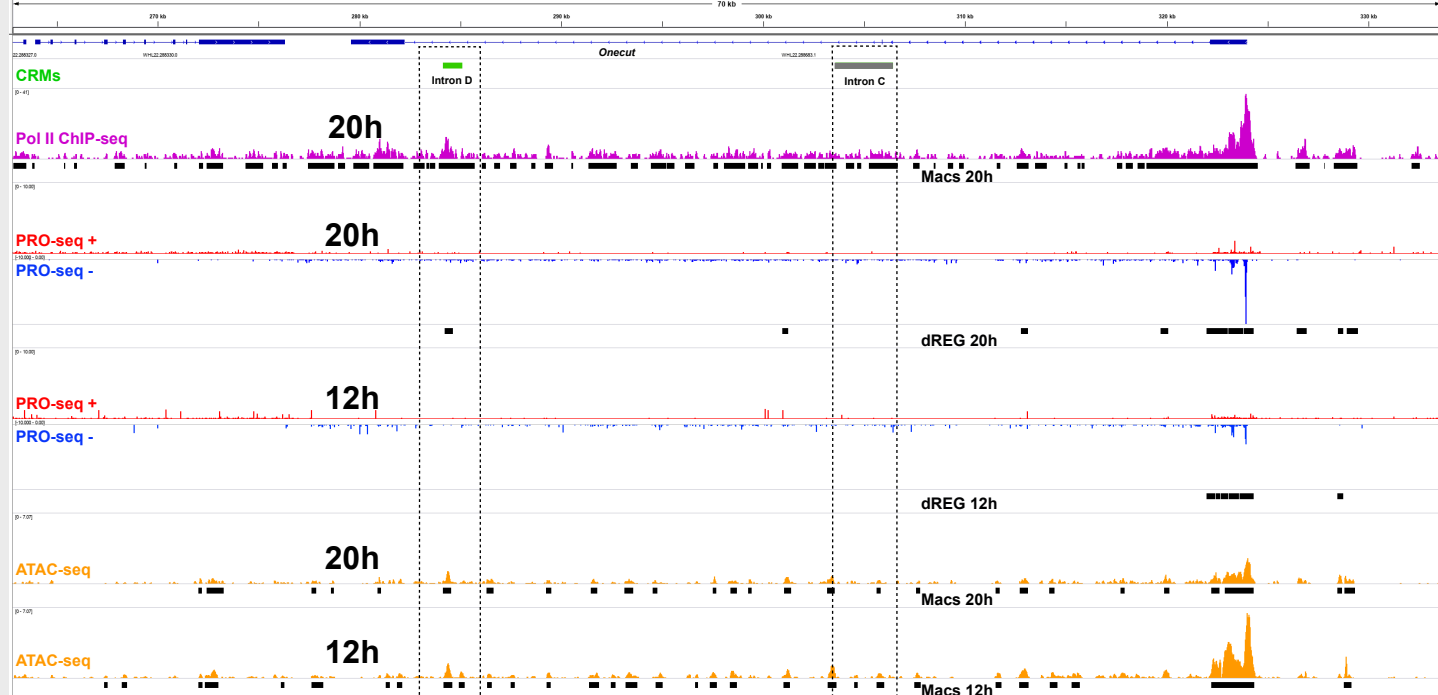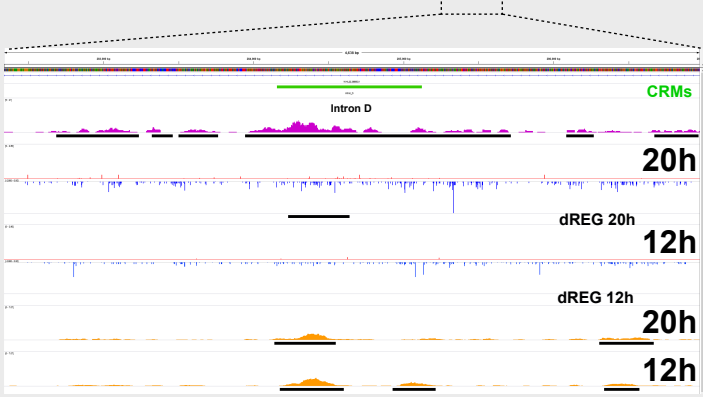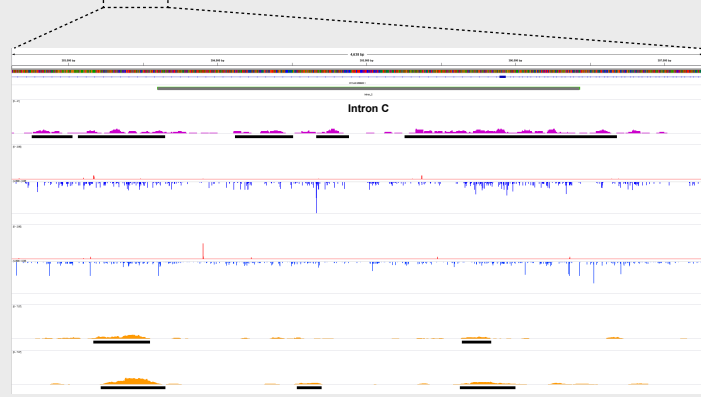

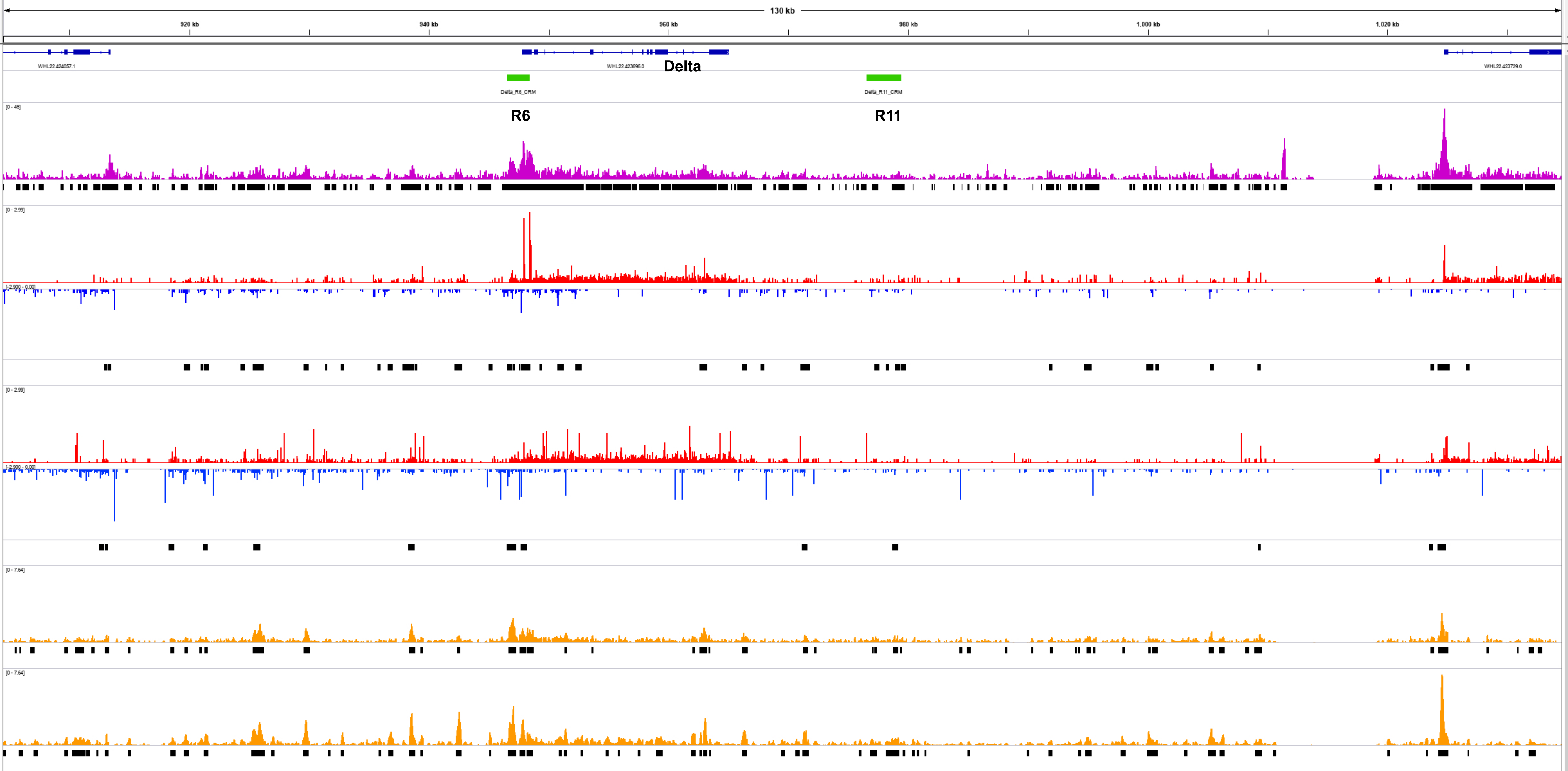

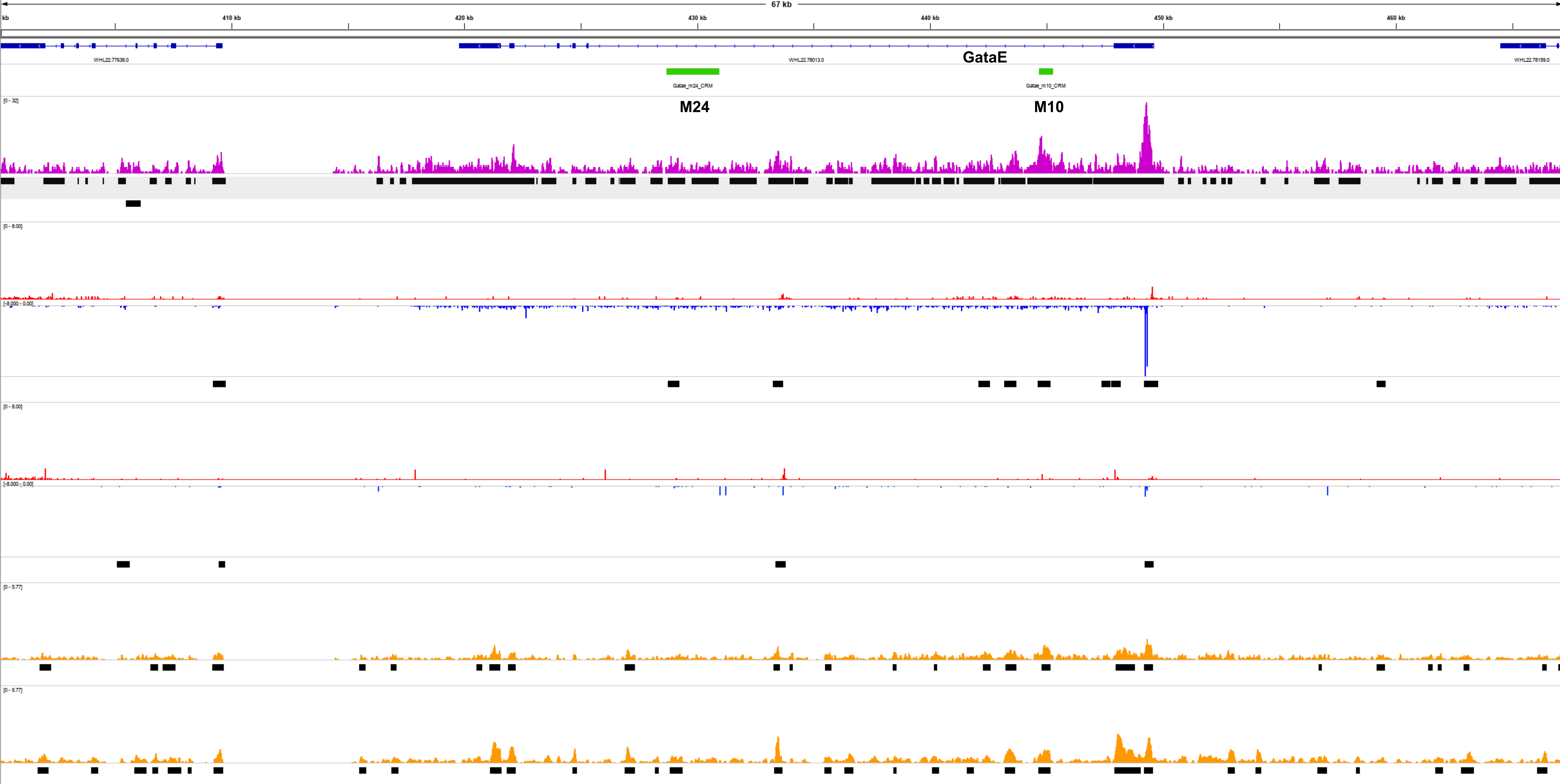

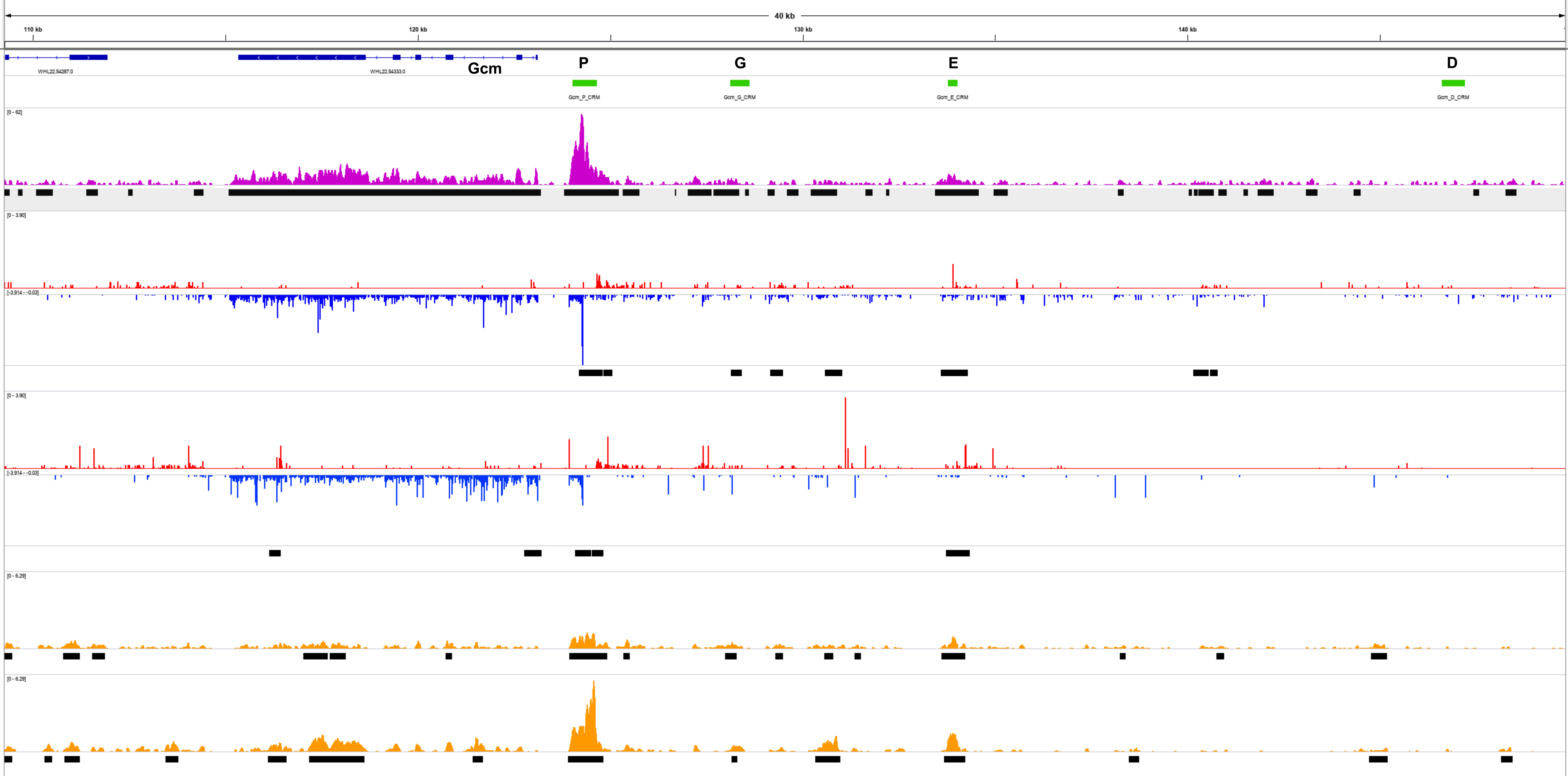

Supplement: Supplementary file 4 — Additional file 4: Fig. S2. PRO-, Pol II ChIP- and ATAC-seq at onecut, gcm, gatae and delta. A, onecut, the entire region was scanned for enhancer activity by overlapping reporter constructs averaging 2.23 Kb [40]. Similar snapshots are taken for other regulatory genes whose regulatory elements were primarily selected based on evolutionary sequence conservation rather than an unbiased tiling scan. [file 12864_2021_7936_MOESM4_ESM.pdf]

**A**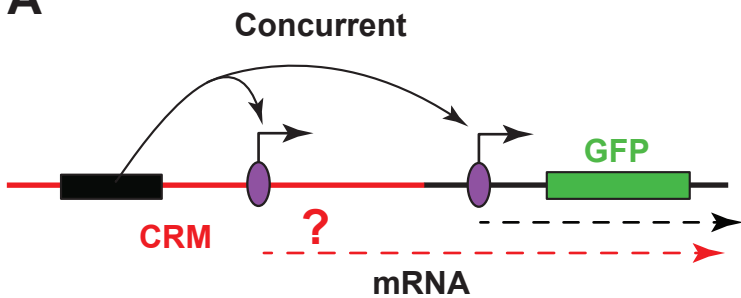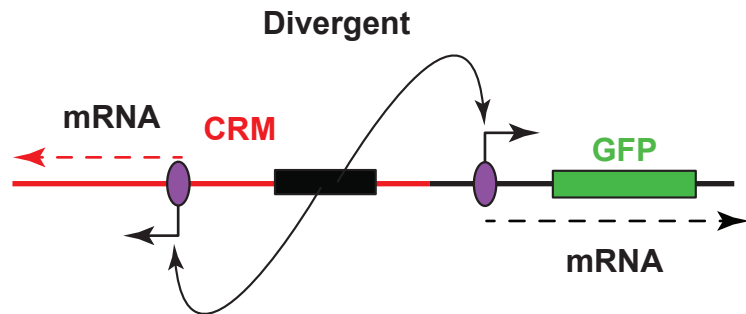**B**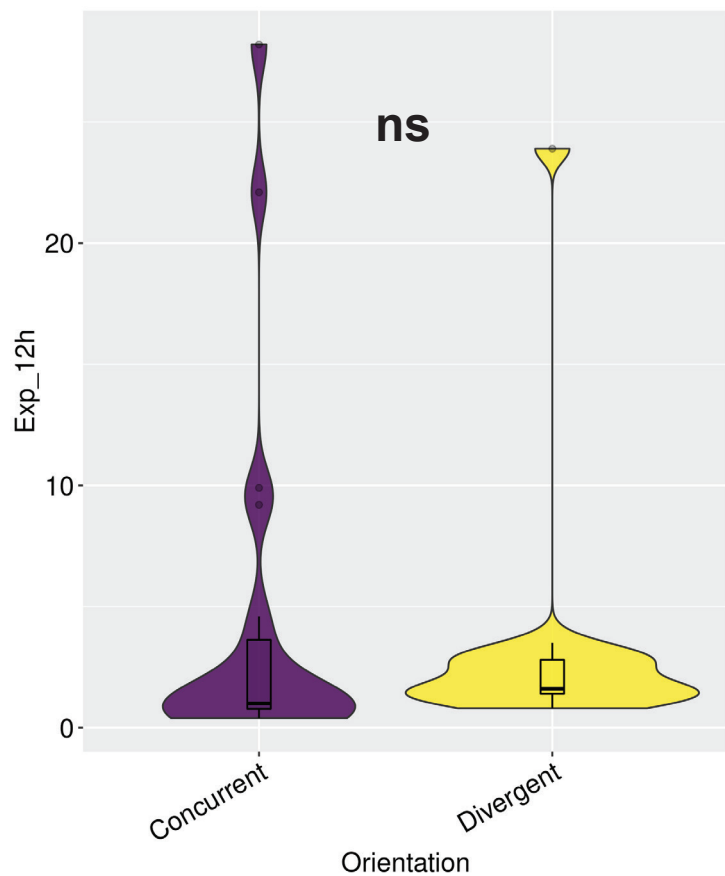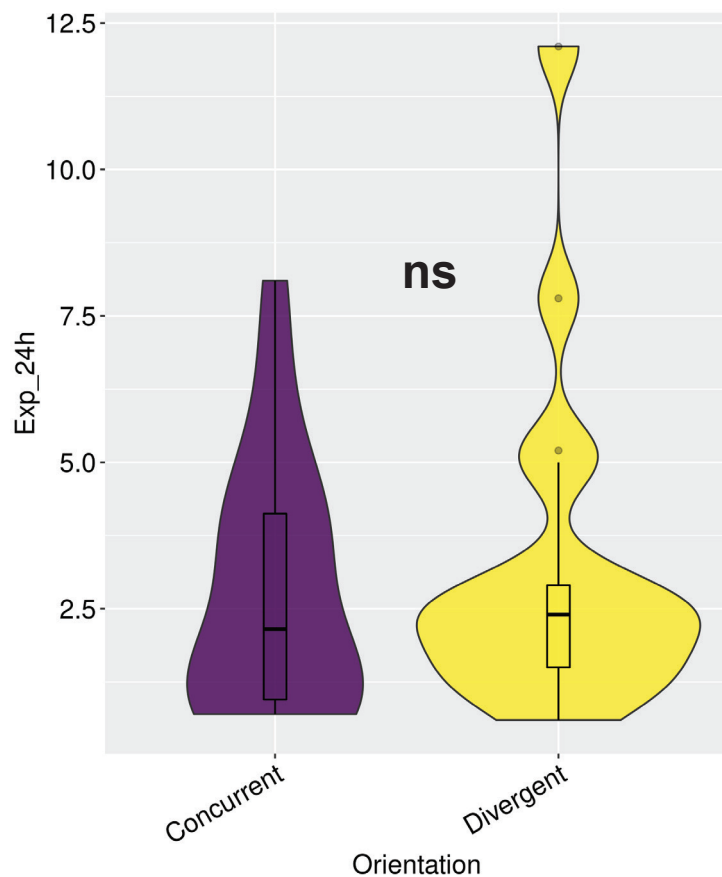

Supplement: Supplementary file 5 — Additional file 5: Fig. S3. The enhancer activity of promoter-overlapping CRMs is independent of their orientation. A, the concurrent orientation of the promoter in the GFP reporter construct and the promoter of promoter-overlapping CRMs could in principle result in transcripts reaching to the coding region of the reporter that could confound CRM driven transcription (red dashed line) with enhancer activity (black dotted line). In both cases the reporter enhancer activity of CRMs containing endogenous promoters should be diminished due to enhancer sharing between both promoters. Correlation of Pol II accumulation at endogenous promoters (purple ovals) and reporter expression is expected for CRMs that contain necessary enhancers and endogenous promoters. B, the difference of expression between CRMs in concurrent and divergent reporter construct orientation is not significant (ns) in 12 and 24 h embryos, Wilcox test p-values of 0.67 and 0.14, respectively. [file 12864_2021_7936_MOESM5_ESM.pdf]

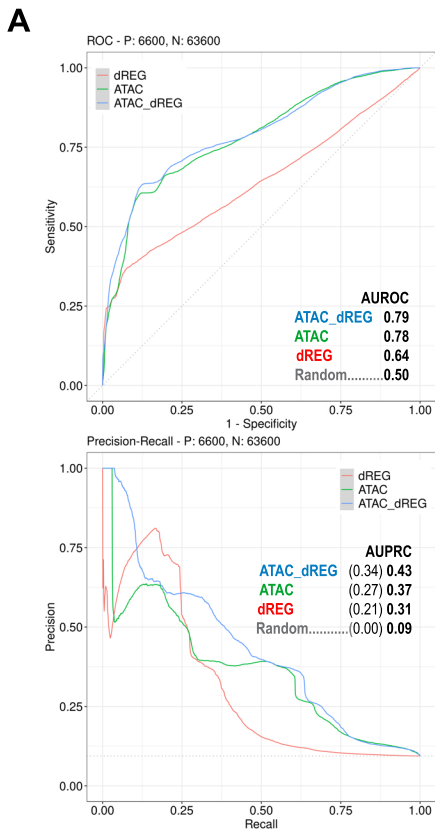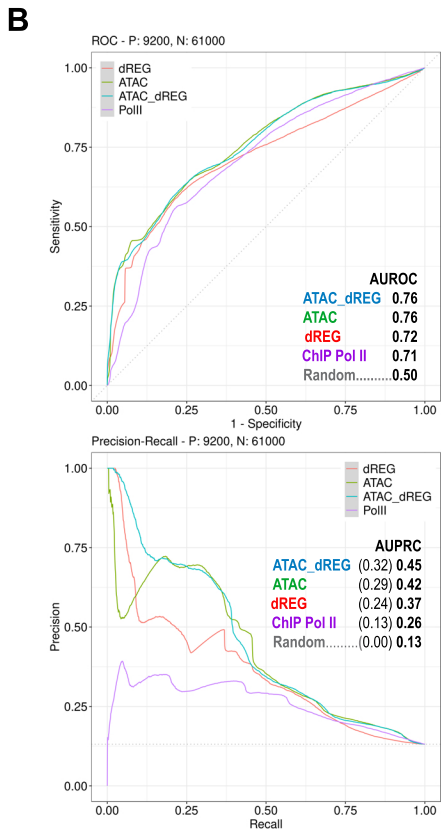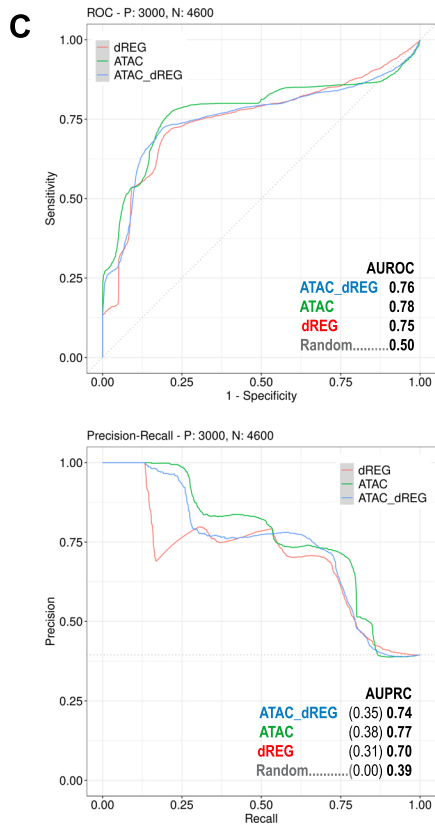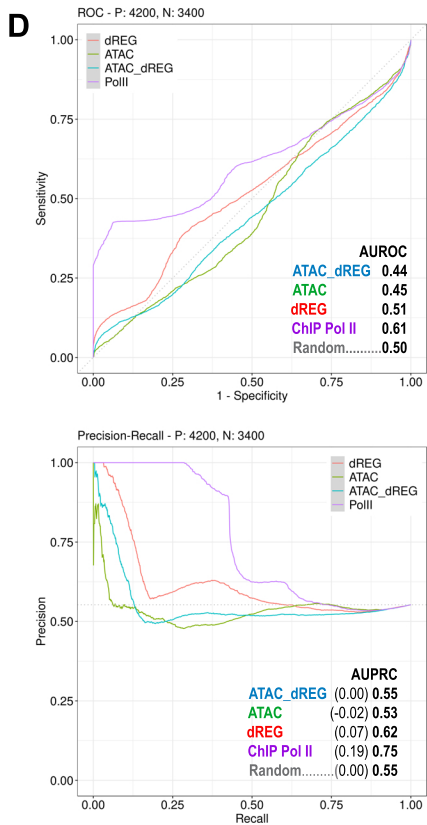

Supplement: Supplementary file 6 — Additional file 6: Fig. S4. Evaluation of models for CRMs overlapping and not overlapping promoters. A, 12 h embryo and, B, 20 h embryo models trained and tested with CRMs not overlapping promoters. C, 12 h embryo and, D, 20 h embryo models trained and tested with CRMs overlapping promoters. [file 12864_2021_7936_MOESM6_ESM.pdf]

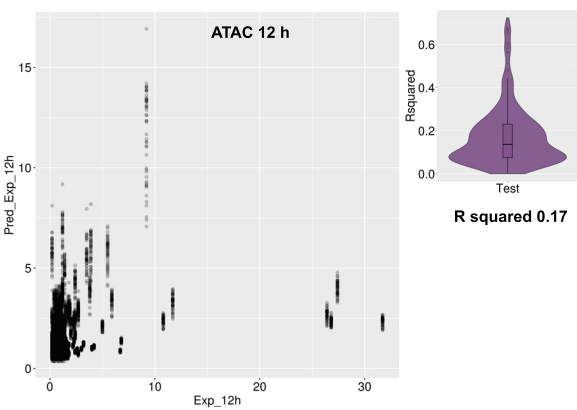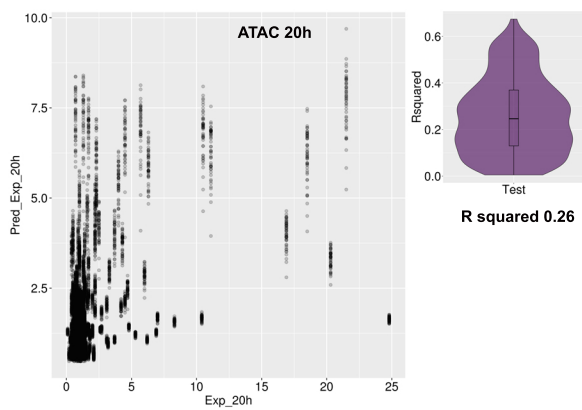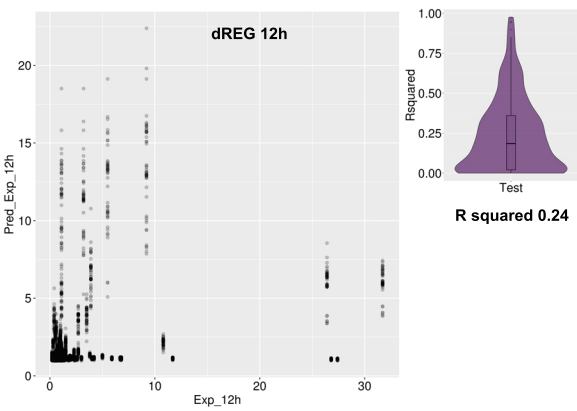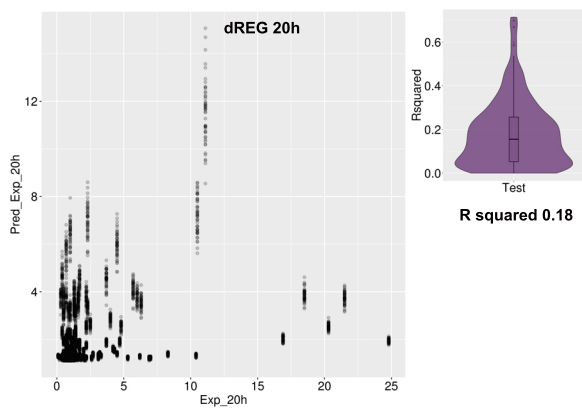

Supplement: Supplementary file 7 — Additional file 7: Fig. S5. Quantitative prediction of enhancer activity from ATAC and PRO-seq data. Plot of the hold-out predicted and actual reporter expression of linear regression models using ATAC and PRO-seq signal in 12 h and 20 h stages. Violin/Box-plots of R2 values, with the average indicated underneath. [file 12864_2021_7936_MOESM7_ESM.pdf]

Log2(20h/12h)

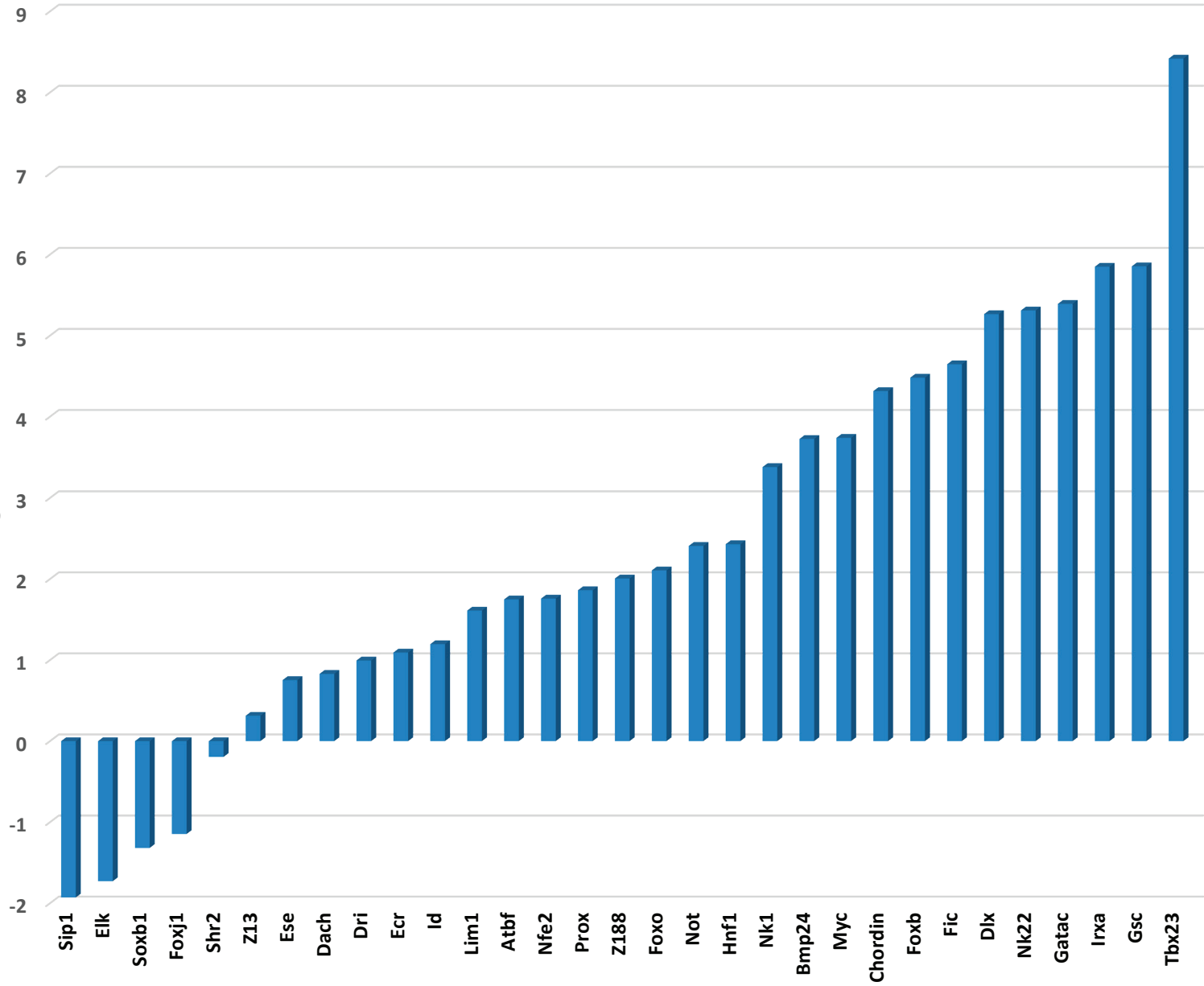

Supplement: Supplementary file 8 — Additional file 8: Fig. S6. Summary 12 to 20 h mRNA expression changes of regulatory genes. The fold change in the mRNA expression levels quantified at high resolution for 31 of the 37 genes used in this study [33]. [file 12864_2021_7936_MOESM8_ESM.pdf]
